# Inhibition of mTOR or MAPK ameliorates *vmhcl/myh7* cardiomyopathy in zebrafish

**DOI:** 10.1172/jci.insight.154215

**Published:** 2021-12-22

**Authors:** Haisong Bu, Yonghe Ding, Jiarong Li, Ping Zhu, Yu-Huan Shih, Mingmin Wang, Yuji Zhang, Xueying Lin, Xiaolei Xu

**Affiliations:** 1 Department of Biochemistry and Molecular Biology, Department of Cardiovascular Medicine, Mayo Clinic, Rochester, Minnesota, USA.; 2 Department of Cardiothoracic Surgery, Xiangya Hospital, Central South University, Changsha, China.; 3 Department of Cardiovascular Surgery, The Second Xiangya Hospital of Central South University, Changsha, China.; 4 Dongzhimen Hospital, Beijing University of Chinese Medicine, Beijing, China.; 5 Department of Epidemiology and Public Health, University of Maryland School of Medicine, Baltimore, Maryland, USA.

**Keywords:** Cardiology, Genetics, Cardiovascular disease, Genetic diseases

## Abstract

Myosin heavy chain 7 *(MYH7)* is a major causative gene for hypertrophic cardiomyopathy, but the affected signaling pathways and therapeutics remain elusive. In this research, we identified ventricle myosin heavy chain like (*vmhcl*) as a zebrafish homolog of human *MYH7*, and we generated *vmhcl* frameshift mutants. We noted *vmhcl*-based embryonic cardiac dysfunction (VEC) in the *vmhcl* homozygous mutants and *vmhcl*-based adult cardiomyopathy (VAC) phenotypes in the *vmhcl* heterozygous mutants. Using the VEC model, we assessed 7 known cardiomyopathy signaling pathways pharmacologically and 11 candidate genes genetically via CRISPR/Cas9 genome editing technology based on microhomology-mediated end joining (MMEJ). Both studies converged on therapeutic benefits of mTOR or mitogen-activated protein kinase (MAPK) inhibition of VEC. While mTOR inhibition rescued the enlarged nuclear size of cardiomyocytes, MAPK inhibition restored the prolonged cell shape in the VEC model. The therapeutic effects of mTOR and MAPK inhibition were later validated in the VAC model. Together, *vmhcl/myh7* loss of function is sufficient to induce cardiomyopathy in zebrafish. The VEC and VAC models in zebrafish are amenable to both efficient genetic and chemical genetic tools, offering a rapid in vivo platform for discovering candidate signaling pathways of *MYH7* cardiomyopathy.

## Graphical abstract

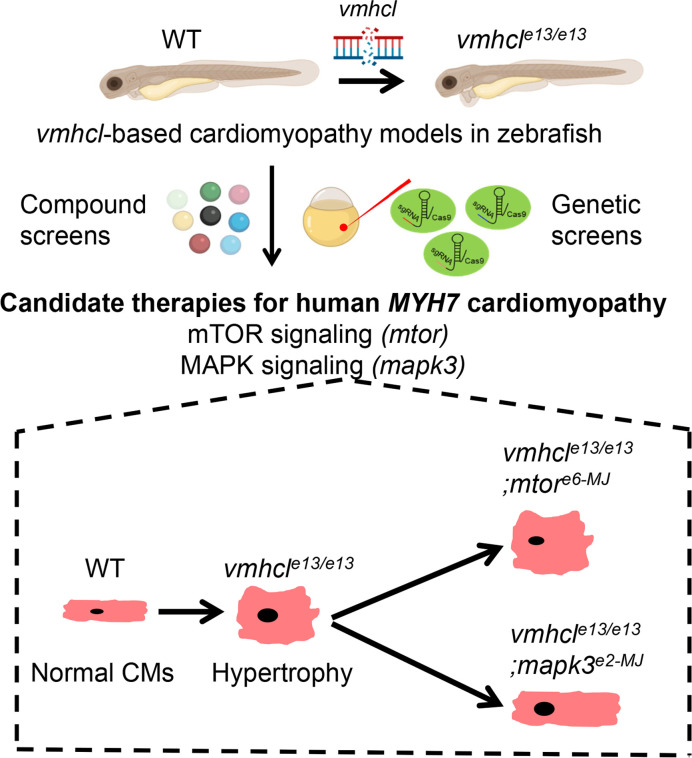

## Introduction

Cardiomyopathy (CM) refers to a group of heterogeneous cardiac muscle diseases that are categorized into hypertrophic CM (HCM), dilated CM (DCM), and restrictive CM (RCM) (1–4). Genetic contributions to CM have been identified, and more than 100 genes have been linked to different types of CM (5, 6). Animal models have been generated and used for discovering key signaling pathways and therapeutic strategies. At least 7 CM signaling pathways with therapeutic potential have been identified, including mitogen-activated protein kinase (MAPK) signaling, mTOR signaling, β-adrenergic receptor signaling, phosphodiesterase 5 (PDE5) signaling, histone deacetylase (HDAC) signaling, Ca^2+^/calmodulin-dependent kinase II signaling, and calcineurin-nuclear factor of activated T cells (Cn-NFAT) signaling pathways (7–9). For example, mTOR is a serine/threonine protein kinase that plays a pivotal role in regulating proteostasis in cardiomyocytes (10–12); partial mTOR inhibition through either pharmacological or genetic approaches exerts cardioprotective effects on several types of cardiomyopathies, including *lamp2*-associated HCM (13), *bag3*-associated and lamin A/C-associated DCM (14, 15), and anemia- and doxorubicin-induced cardiomyopathies (DICs) (16). In contrast, MAPKs have been found to activate in response to almost every stress- and agonist-induced hypertrophic stimulus, and uniquely regulate the balance between eccentric and concentric growth of the heart (17, 18). Whereas activation of MAPK results in eccentric hypertrophy and promotes myocyte lengthening, inhibition of the extracellular signal–regulated kinase (ERK) pathway results in an attenuated hypertrophic response to pressure overload (19).

*MYH7,* also termed *β*-*myosin heavy chain*, was the first identified CM-causative gene, which later was determined to be responsible for ~18% of HCM cases (20–22). In humans, *MYH7* is located in tandem with *MYH6* on chromosome 14, with *MYH7* being the predominant adult isoform that is located upstream of *MYH6*. In mice, *Myh7* and *Myh6* are also located in tandem on chromosome 14; however, the upstream *Myh7* gene is mainly expressed in the embryonic heart, whereas *Myh6* is expressed in the adult heart (23), an opposite pattern to that in humans. Mechanistic studies of *MYH7* R403Q, which causes a common and particularly malignant form of inherited HCM (24), yield conflicting information from studies in mice versus those in large mammals/humans. Earlier results with human cardiac myosin showed a loss of function (25, 26), which is supported by recent transgenic studies in rabbits carrying an R403Q mutation in *MYH* (22, 27, 28). In contrast, transgenic studies in mice suggested a gain-of-function nature of *Myh7* R403Q (25, 29). Whether any known CM pathways are effective at ameliorating *MYH7* CM remains untested, to our knowledge.

Zebrafish (*Danio rerio*) is an emerging vertebrate model for CMs, and *titin* and *troponin T* mutants were considered as the first embryonic CM models (30, 31). However, it remains unclear whether these embryonic models faithfully recapitulate pathogenesis in human CMs, a group of progressive diseases with late onset. To address this concern, adult zebrafish CM models have been developed, such as those induced by anemia, doxorubicin, and diabetes (32). The advent of genome editing technology enabled the generation of inherited CM models with mutations in known causative genes, such as *titin* (33, 34), *bag3* (14), and *lamp2* (13). Compared to rodents, zebrafish are amenable to more efficient genetics and chemical genetic tools. A CRISPR/Cas9 genome editing technology based on microhomology-mediated end joining (MMEJ) was recently established, and this approach has enabled the generation of predictable biallelic loss-of-function mutants in F0 fish to allow a rapid establishment of genotype-phenotype relationships (35). Owing to their high throughput and excellent permeability, zebrafish embryos have also been successfully utilized to conduct pharmacological screens and identify compounds with therapeutic capacity for doxorubicin-induced cardiotoxicity and the induction of *anf*, a molecular marker of CM (36, 37). Moreover, a mutagenesis screen–based strategy has been established to discover novel genetic factors and therapeutic target genes for CMs (3, 38, 39).

Encouraged by these powerful genetic and chemical genetic capabilities, we explored zebrafish as an alternative animal model for *MYH7* CM. Utilizing transcription activator–like effector nuclease (TALEN) technology, we generated frameshift mutants for the *ventricle myosin heavy chain-like* (*vmhcl*) gene, also known as *myh7l*. We provided evidence of its identity as an *MYH7* homolog and established both *vmhcl* homozygous embryos and heterozygous adults as models for *MYH7* CM. By performing both compound testing and genetic assessments using the MMEJ-based CRISPR/Cas9 genome editing technology in the embryonic model, we identified the therapeutic effects of mTOR and MAPK inhibition. Our data established zebrafish as an in vivo model for mechanistic study and therapeutic development for *MYH7* CM.

## Results

### Depletion of vmhcl, a candidate MYH7 homolog, induces cardiac dysfunction in zebrafish embryos.

Our previous transcriptome studies identified 9 *myh* homologs in the zebrafish genome (40), among which atrial myosin heavy chain (*amhc*), ventricular myosin heavy chain (*vmhc*), and ventricular myosin heavy chain-like (*vmhcl*) are 3 predominant cardiac homologs, accounting for more than 99% of transcripts in the zebrafish heart. The *vmhcl* gene encodes a protein that shares 85.9% similarity with the human *MYH7* protein (Supplemental Figure 1; supplemental material available online with this article; https://doi.org/10.1172/jci.insight.154215DS1). Similar to *vmhc* but not *amhc*, *vmhcl* is specifically expressed in the ventricle, as indicated by in situ whole-mount staining (Supplemental Figure 2, A and B). Consistent with our transcriptome data indicating that *vmhc* is a predominant embryonic *myh* isoform and *vmhcl is* a predominant adult isoform (40), we found that the expression of the *vmhc* transcripts gradually decreased after 10 weeks of age and were undetectable after 13 weeks; in contrast, cardiac expression of *vmhcl* transcripts remained strong until at least 13 weeks (Supplemental Figure 2C). In zebrafish, *vmhcl* and *vmhc* are located in tandem on chromosome 2, with *vmhcl* located approximately 4.4 kb upstream of *vmhc* (Supplemental Figure 2D) (40). Because of the similarity with *MYH7/MYH6* in humans, where the upstream gene is the predominant adult cardiac isoform and the downstream gene is mainly expressed in embryonic hearts (40), we propose that *vmhcl* is an *MYH7* homolog in zebrafish and *vmhc* is an *MYH6* homolog.

**Figure 1:**
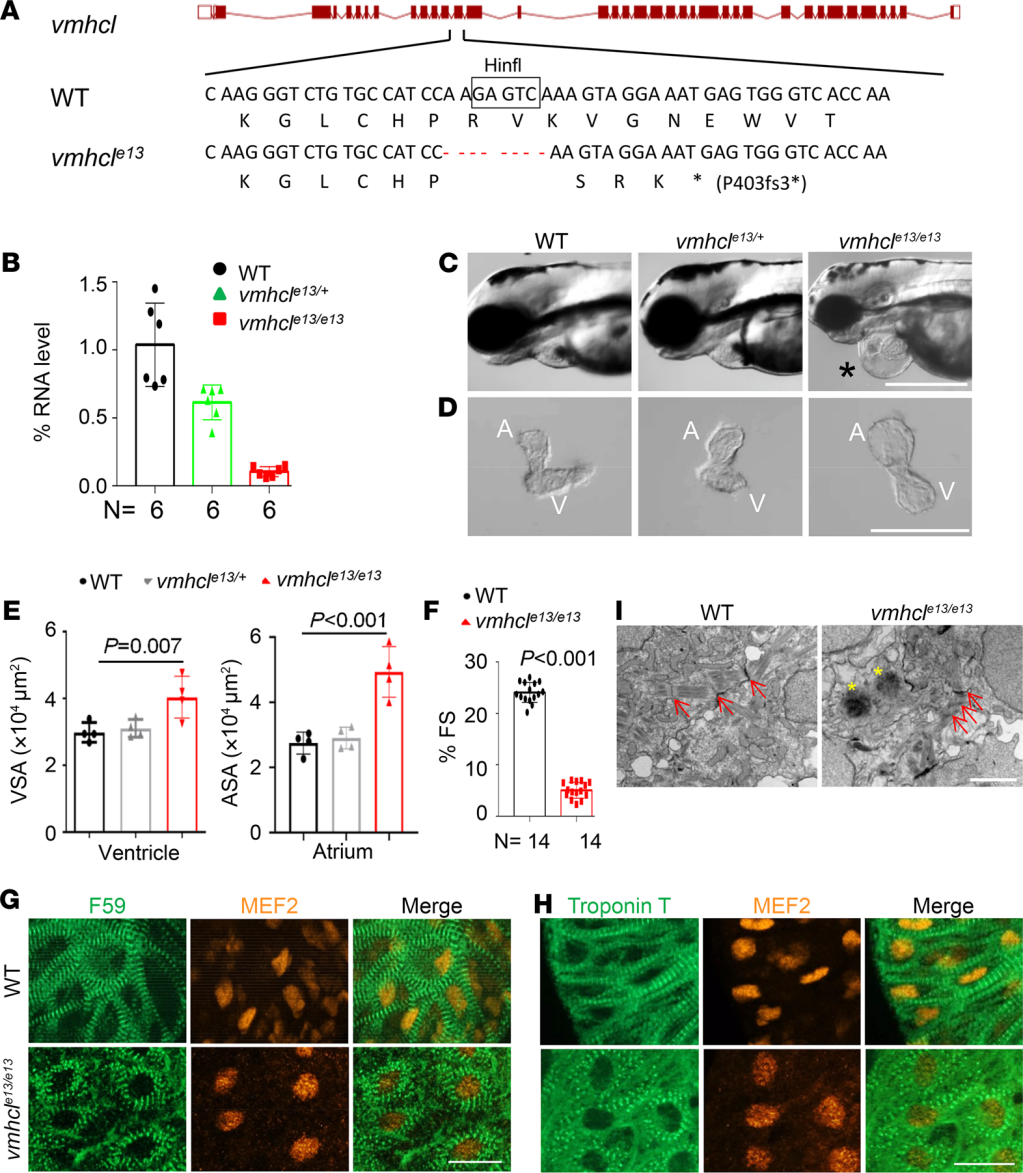
Depletion of *vmhcl* disrupts sarcomere integrity and results in a VEC model. (**A**) Schematics of the *vmhcl* mutant allele generated using TALEN. The Hinfl restriction enzyme recognition site used for genotyping is boxed. Dashed lines indicate deleted nucleotides. The asterisk indicates an early translational stop codon. fs, frameshift. (**B**) Quantitative reverse transcription PCR (RT-PCR) revealed reduced expression of the transcript in both heterozygous (*vmhcl^e13/+^*) and homozygous (*vmhcl^e13/e13^*) mutants. (**C**–**E**) Representative images and quantification of the VSA and atrial surface area (ASA) in fish at 3 dpf. The asterisk indicates edema. A, atrium; V, ventricle; *n* = 4; 1 way ANOVA. (**F**) Percent FS of the *vmhcl^e13/e13^* mutant compared to WT control at 3 dpf. *n* = 14; data are presented as the mean ± SD. Paired 2-tailed Student’s *t* tests were used for statistical analyses. (**G** and **H**) The *vmhcl^e13/e13^* mutants exhibited disrupted sarcomere structure at 3 dpf. Fluorescence immunostaining using anti-myosin heavy chain 1 (F59) and anti-troponin T antibodies are shown. (**I**) Transmission electron microscope (TEM) images confirmed the disrupted sarcomere structure in the *vmhcl^e13/e13^* mutants. Arrows indicate sarcomeric Z-discs in the *vmhcl^e13/e13^* mutant compared to WT control. Asterisks point to degenerated sarcomere in the *vmhcl^e13/e13^* mutant. Scale bars: 500 μm in **C**, 200 μm in **D**, 10 μm in **G** and **H**, and 2 μm in **I**.

We then generated *vmhcl^e13^*, a *vmhcl* mutant harboring an 8-nucleotide deletion (Figure 1A and Supplemental Figure 3) by targeting the 13th exon of *vmhcl* using TALEN technology. The expression of the *vmhcl* transcript was reduced by 39% in *vmhcl^e13/+^* and 90% in *vmhcl^e13/e13^* zebrafish (Figure 1B), likely due to nonsense-mediated RNA decay. The *vmhcl^e13/e13^* homozygous mutants manifested pericardial edema starting at 3 days after fertilization (dpf) (Figure 1C), concurrent with significantly enlarged chamber size (Figure 1, D and E). The cardiac pump function, as measured by fractional shortening (FS), was significantly reduced in the *vmhcl^e13/e13^* homozygous hearts as well (Figure 1F). Because *vmhcl* encodes a sarcomeric protein, we assessed the sarcomere structure in *vmhcl^e13/e13^* zebrafish. Striated thick and thin filaments were disrupted in the homozygous mutants, as revealed by immunostaining using an anti–myosin heavy chain 1 A (MYH1A) (F59) or anti–troponin T antibody, respectively (Figure 1, G and H). These sarcomeric phenotypes were confirmed by transmission electronic microscopy (TEM) analysis (Figure 1I). Consistent with the ventricle-specific expression pattern of *vmhcl*, these sarcomeric abnormalities occurred only in the ventricle but not in the atrium (Supplemental Figure 4, A, B, and D). The enlarged atrium size (Figure 1E), manifesting as reduced cardiomyocyte cell density and increased cardiomyocyte cell size (Supplemental Figure 4), was considered to be a secondary effect of ventricule dysfunction. Taken together, these results suggest that depletion of *vmhcl* yields a *vmhcl*-based embryonic cardiac dysfunction (VEC) model.

### vmhcl haploinsufficiency results in CM-like phenotypes in adult zebrafish.

**Figure 2:**
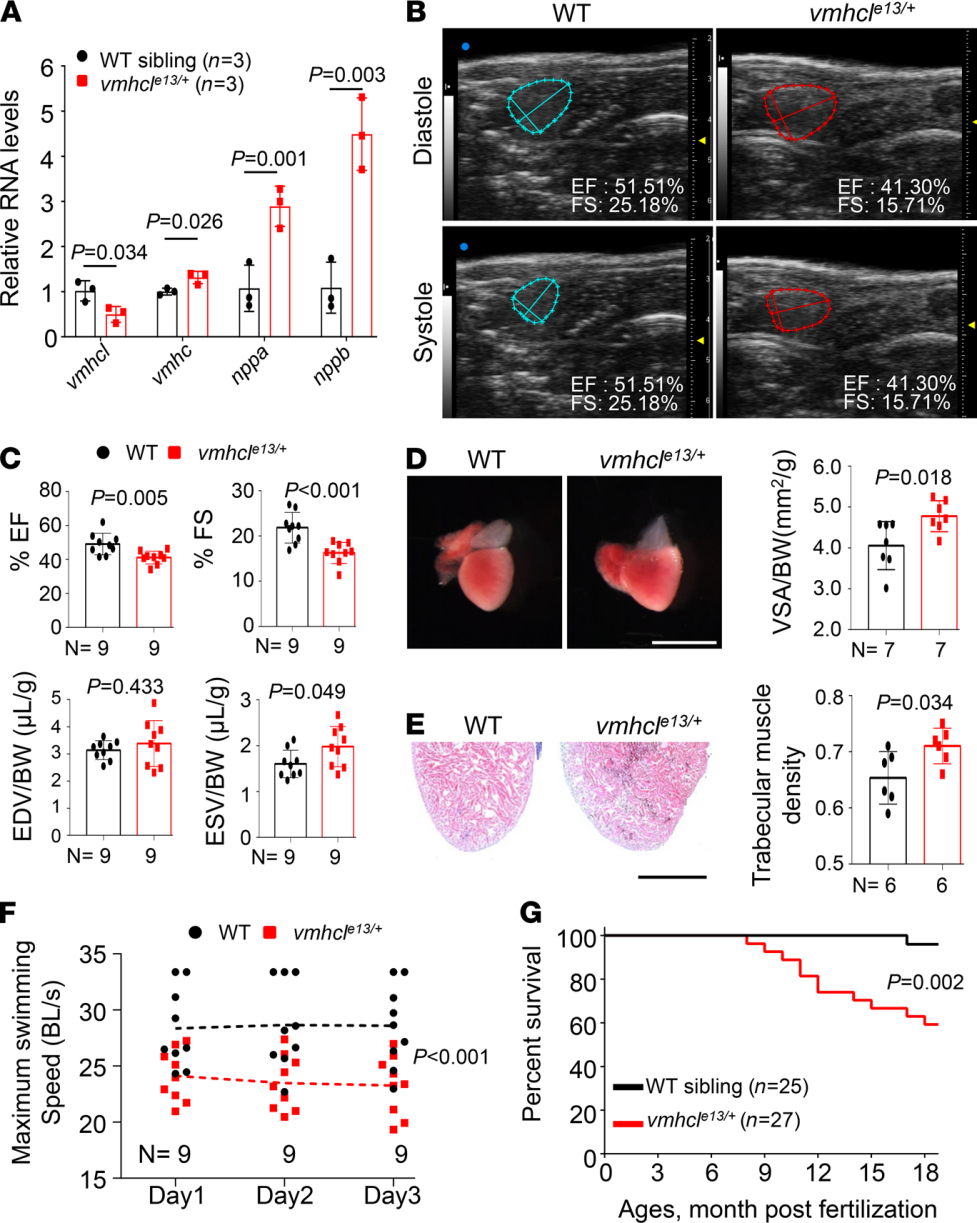
Haploinsufficiency of *vmhcl* results in CM in adult zebrafish. (**A**) Quantitative RT-PCR analysis of CM molecular markers. *n* = 3 biological replicates; 2 tailed Student’s *t* test. (**B**) Representative echocardiography images of WT controls and *vmhcl^e13/+^* mutants at diastole and systole. (**C**) Quantification of cardiac function indices measured using echocardiography in the *vmhcl^e13/+^* mutant and WT control at 8 months. *n* = 9; data are presented as the mean ± SD; unpaired 2 tailed Student’s *t* test. (**D**) Representative images of isolated hearts and quantification of the VSA normalized to the BW of fish at 8 months. *n* = 7; unpaired 2 tailed Student’s *t* test. (**E**) Representative images of H&E staining in the apex area and quantification of trabecular muscle density in fish at 8 months. *n* = 6; unpaired 2 tailed Student’s *t* test. (**F**) Maximum swimming speed of fish at 8 months. *n* = 9; 2-way ANOVA. (**G**) Kaplan–Meier survival curves of *vmhcl* mutant fish and WT controls. *n* = 25–27; log-rank test; data are presented as the mean ± SD. Scale bars: 2 mm in **D** and 300 μm in **E**.

Next, we studied the *vmhcl^e13/+^* fish. At the protein level, the levels of *Myh* bands of approximately 200 kDa in size were reduced in the *vmhcl^e13/+^* mutant at 6 months (Supplemental Figure 5, A and B). At the mRNA level, *vmhcl* haploinsufficiency manifested as reduced *vmhcl* transcripts that were compensated by increased expression of the *vmhc* and *amhc* transcripts, without affecting the expression of *myh7ba* and *myh7bb*, 2 minor zebrafish cardiac *myh* homologs (Figure 2A and Supplemental Figure 5C). We noted significant decreases in the ejection fraction (EF) and FS, as measured by high frequency echocardiography, in the *vmhcl^e13/+^* mutant zebrafish at 8 months (Figure 2, B and C). The reduction in cardiac pump function was largely ascribed to the increased end-systolic volume/body weight (ESV/BW) ratio, while the end-diastole volume/BW (EDV/BW) ratio remained unchanged. Consistent with this observation, we noted an enlarged heart chamber size, as indicated by the significantly increased ventricular surface area/BW (VSA/BW) index in the *vmhcl^e13/+^* mutant hearts (Figure 2D). We also noted increased trabecular muscle density in the sectioned *vmhcl^e13/+^* fish hearts, as determined by H&E staining analysis (Figure 2E). At the molecular level, the expression of cardiac remodeling markers, such as natriuretic peptide B (*nppb*) and natriuretic peptide A (*nppa*), was significantly elevated in the *vmhcl^e13/+^* mutant fish (Figure 2A). Exercise capacity, an important clinical index of heart failure in human patients, was also compromised in the *vmhcl^e13/+^* mutant fish (Figure 2F). The *vmhcl^e13/+^* mutant fish started to die at 8 months old, and only approximately 60% of fish survived to 1.5 years of age (Figure 2G). In summary, the adult *vmhcl^e13/+^* zebrafish manifested CM-like phenotypes and were designated as a *vmhcl*-based adult CM (VAC) model.

### Compound and genetic screens in the VEC model identified mtor and mapk3 as candidate modifier genes.

**Figure 3:**
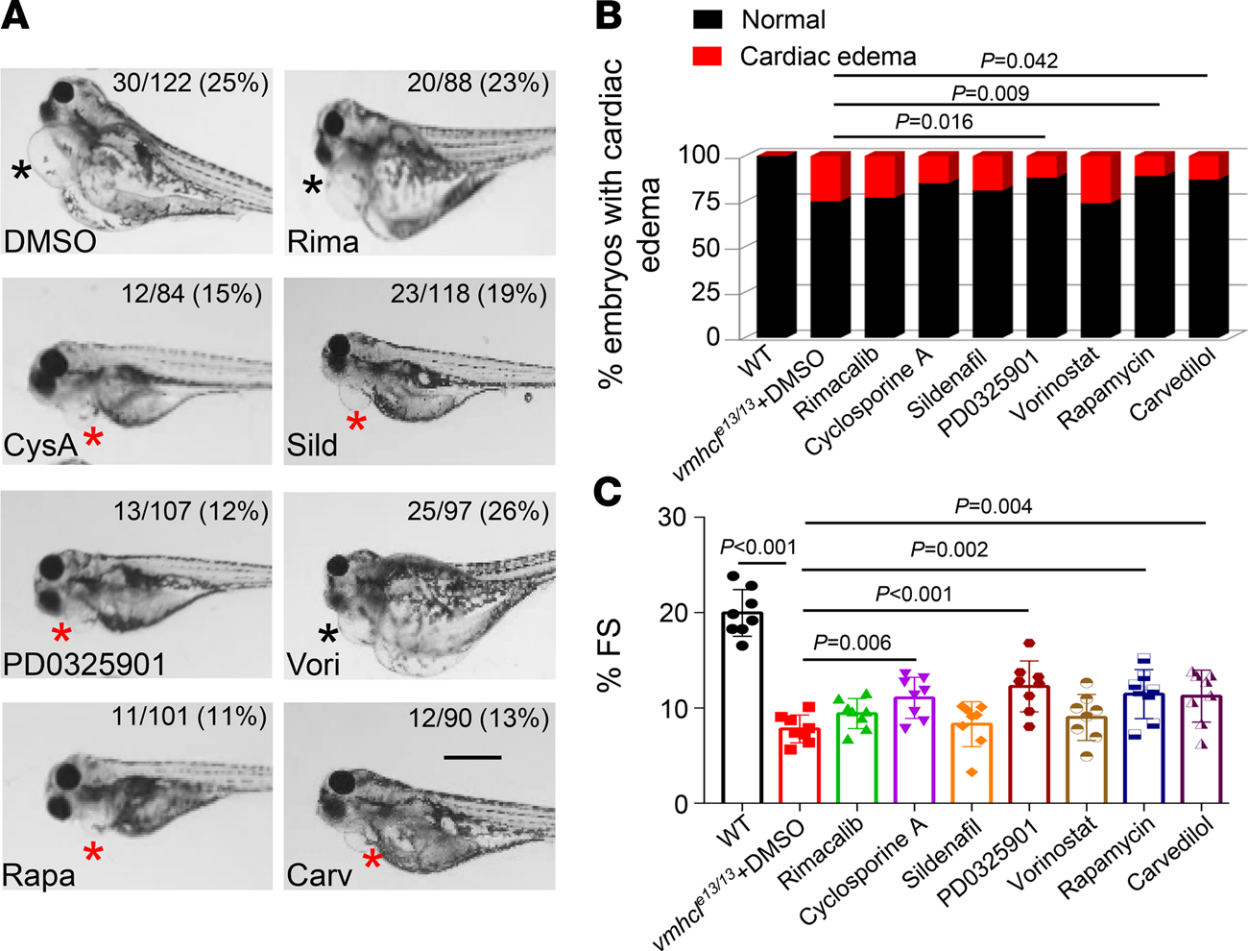
A screen of compounds targeting 7 known CM pathways identified therapeutic compounds for VEC. (**A** and **B**) Representative images and percentage of *vmhcl^e13/e13^* mutant fish with the indicated phenotypes after administration of the compounds or DMSO control at 5 dpf. Black stars indicate severe edema, and red stars indicate mild edema. It is anticipated that 25% of *vmhcl^e13/+^* incross offspring are homozygous *vmhcl^e13/e13^*, which manifest severe pericardial edema (black stars). Administration of 5 different drugs reduced the percentage of *vmhcl^e13/e13^* mutants with edema to less than 20%; 3 of these drugs reduced the percentage with edema with statistical significance (*P* < 0.05). *n* = 84–122; χ^2^ test; scale bar: 500 μm. (**C**) Percent FS of the *vmhcl^e13/e13^* mutants after administration of the compounds compared to WT control at 5 dpf. *n* = 8; data are presented as the mean ± SD; 1-way ANOVA.

To explore the use of the VEC model for identifying signaling pathways with therapeutic potential, we assessed small-molecule inhibitors of 7 known CM signaling pathways (Table 1) (7). Effective doses of these small-molecule inhibitors were determined to avoid toxic effects (38, 41–44). At 5 dpf, 3 of 7 inhibitors (PD0325901, rapamycin, and carvedilol) mitigated the phenotypes in *vmhcl^e13/e13^* zebrafish, as indicated by significantly reduced percentages of homozygous *vmhcl^e13/e13^* fish with severe pericardial edema (Figure 3, A and B, and Supplemental Figures 6 and 7), partially restored cardiac function (Figure 3C), and survival (Supplemental Figure 6B). Although none of these inhibitor treatments allowed homozygous *vmhcl^e13/e13^* zebrafish to survive to adulthood (Supplemental Figure 6B), the transient modulation of cardiac edema severity and cardiac function suggests that CM pathways can be studied in the VEC model.

**Figure 4:**
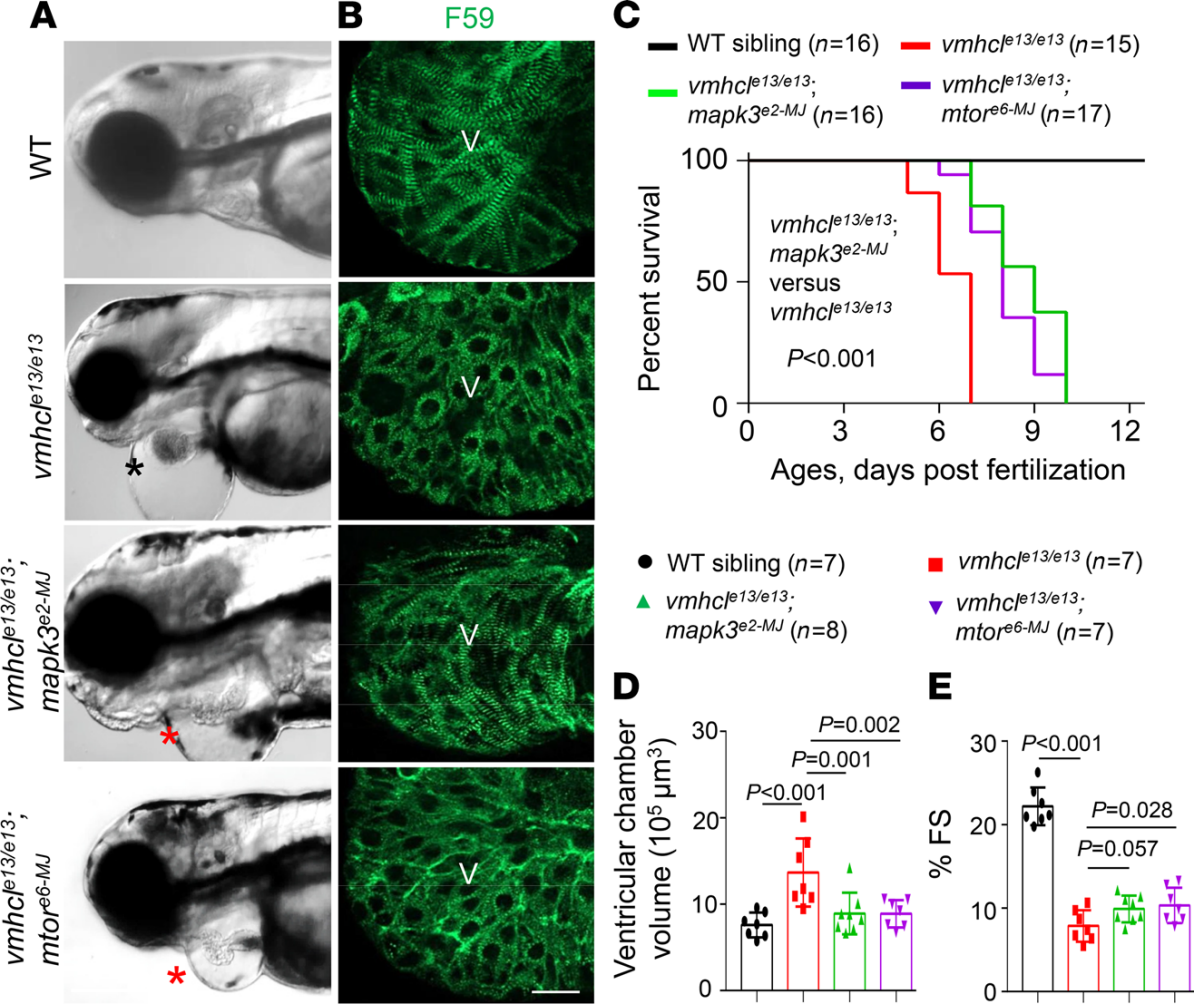
An F0 screen of 11 target genes in 7 known CM pathways identified *mapk3* and *mtor* as 2 therapeutic modifier genes for VEC. (**A**) Representative images of the heart area of F0 fish at 3 dpf. The black star indicates severe edema, and red stars indicate mild edema. Scale bar: 300 μm. (**B**) Fluorescence immunostaining images using anti-myosin heavy chain 1 (F59) in the ventricles of (from top to bottom): WT controls, *vmhcl^e13/e13^*, *vmhcl^e13/e13^;mapk3^e2-MJ^*, and *vmhcl^e13/e13^;mtor^e6-MJ^* mutant hearts at 3 dpf. Scale bar: 2 μm; V, ventricle. (**C**) Kaplan–Meier survival curves of *vmhcl^e13/e13^* mutant fish upon *mapk3* and *mtor* inhibition and WT controls. *n* = 15–17; log-rank test. (**D** and **E**) VCV (**D**) and percent FS (**E**) of the *vmhcl^e13/e13^* mutants after *mapk3* and *mtor* inhibition compared to WT controls at 3 dpf. *n* = 7–8; data are presented as the mean ± SD; 1-way ANOVA.

Encouraged by our success with a compound-based pathway assessment (38), we then analyzed these signaling pathways genetically using the MMEJ-based CRISPR/Cas9 genome editing technology that enables rapid establishment of geneotype-phenotype relationships in F0 fish (45, 46). We selected 11 target genes from 7 known CM signaling pathways that could exert therapeutic effects on certain types of CM (Table 2) (7, 8), designed MMEJ-based sgRNAs for these genes, injected different doses of sgRNAs into zebrafish embryos at the 1-cell stage, and optimized doses by measuring resultant KO scores (Table 2 and Supplemental Table 1). We then injected sgRNAs into the offspring of *vmhcl^e13/+^* incrosses. We found that injection of sgRNAs for the *mtor* or *mapk3* genes, but not the other 9 target genes, mitigated cardiac phenotypes in the *vmhcl^e13/e13^* homozygous mutant, as indicated by rescuing effects on cardiac edema (Figure 4A), survival (Figure 4C), ventricular chamber volume (VCV) (Figure 4D), and percent FS as well (Figure 4E). Notably, the rescuing effects of *mtor* inhibition on FS were marginal; injection of sgRNAs for the *mapk3*, but not the *mtor*, also partially restored the disrupted sarcomere structure in the *vmhcl^e13/e13^* homozygous mutant (Figure 4B).

To confirm these observations from transient genetic studies, we then generated the *vmhcl^e13/e13^mtor^xu015/+^* double stable mutants, by crossing the *vmhcl^e13/+^* with *mtor^xu015/+^* (16). Similar to the transient injection with the *mtor* MMEJ sgRNA, *mtor* haploinsufficiency rescued the phenotypes of *vmhcl^e13/e13^* fish, as indicated by the rescued VCV and increased survival (Supplemental Figure 8, A, B, and D). Similar to genetic studies in F0, the rescuing effects on cardiac function were not significant (Supplemental Figure 8C). To generate stable mutants for *mapk3*, we raised F0 fish injected with a *mapk3* targeting sgRNA to adults, selected fish predominantly harboring the 4 bp nucleotide deletion in their genome (Supplemental Figure 9, A and B), and incrossed them to obtain F1 offspring. We identified F1 fish harboring a stable *mapk3* mutation and designated these fish as *mapk3^e2-F1^*. Consistent with our genetic analysis in F0, we detected therapeutic effects of *mapk3^e2-F1^* on the *vmhcl^e13/e13^* phenotype, as evidenced by significantly reduced VCV and improved cardiac function in *vmhcl^e13/e13^mapk3^e2-F1^* fish (Supplemental Figure 9, C–E).

### Distinct cellular effects of mtor inhibition and mapk3 inhibition on the VEC model.

**Figure 5:**
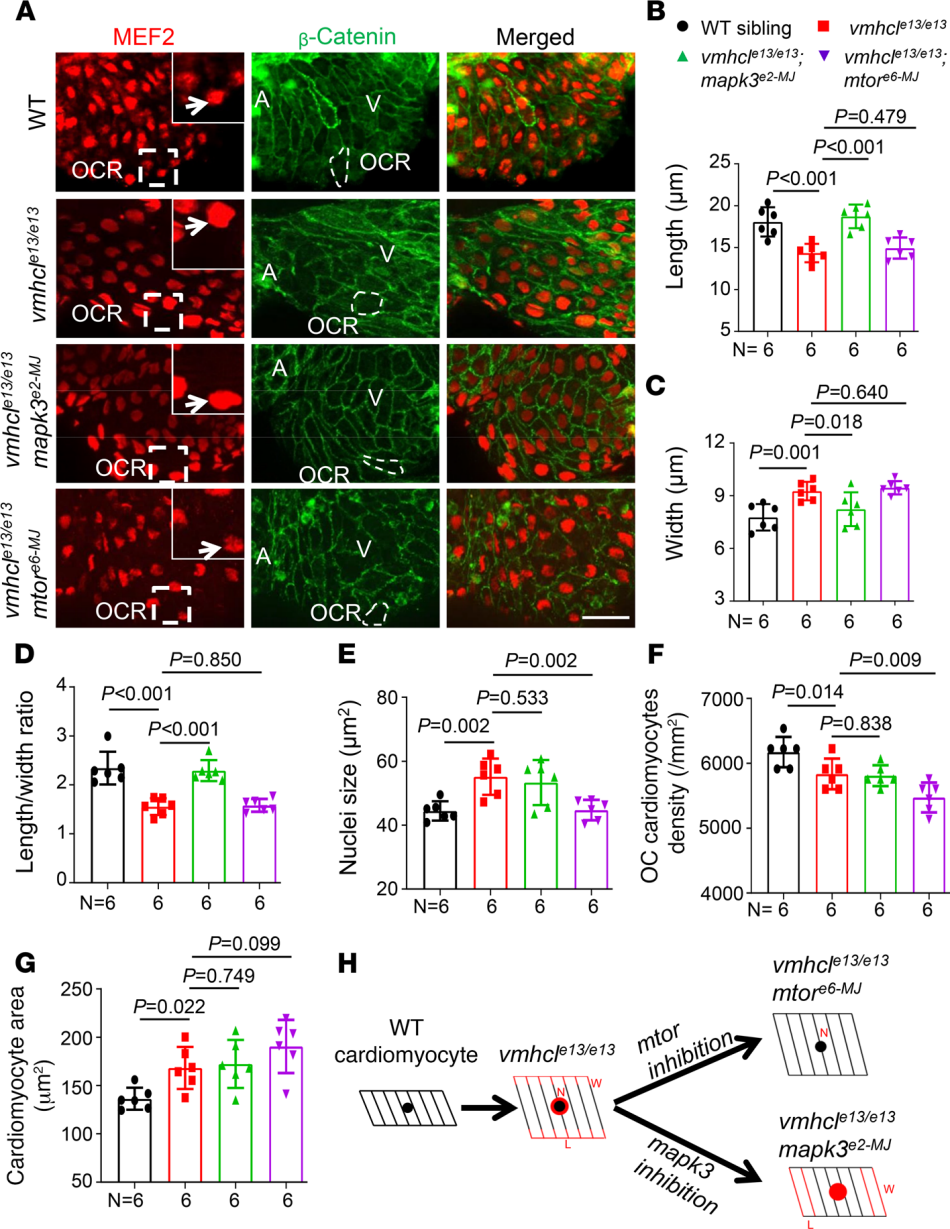
Distinct cellular effects of *mtor* and *mapk3* inhibition on the VEC model. (**A**) Representative images of the nucleus and outline of cardiomyocytes were obtained by immunostaining with anti–myocyte enhancer factor-2 (red) and anti–β-catenin (green) antibodies in WT controls and *vmhcl^e13/e13^*, *vmhcl^e13/e13^mapk3^e2-MJ^*, and *vmhcl^e13/e13^mtor^e6-MJ^* mutants at 3 dpf. The insets show the boxed areas at higher original magnification. The white arrows indicate nuclei of the cardiomyocytes. Representative cardiomyocytes in the OCR are outlined by dashed white lines in panels with β-catenin staining. A, atrium; V, ventricle; scale bar: 25 μm. (**B**–**G**) Quantification of the cardiomyocyte length (**B**), cardiomyocyte width (**C**), cardiomyocyte length/width ratio (**D**), cardiomyocyte nuclei size (**E**), cardiomyocyte density (**F**), and cardiomyocyte area (**G**) measured in these mutants and WT controls. *n* = 6; data are presented as the mean ± SD; 1-way ANOVA. (**H**) Schematics of different cellular changes observed upon *mapk3* and *mtor* inhibition in the VEC model. Red indicates changes in cardiomyocyte length (L), width (W), or nuclei size (N).

To discern the functions of *mtor* inhibition and *mapk3* inhibition, we examined cellular changes in the VEC model. Using an anti–β-catenin antibody to define the cell border and an anti–myocyte enhancer factor-2 (anti-Mef2) antibody to label the cardiomyocyte nuclei (Figure 5A), we noted a marked change in cardiomyocyte morphometry in the outer curvature (OCR) of the ventricles in the VEC model at 3 dpf. Compared to a prolonged shape in wild-type cardiomyocytes, these mutant cardiomyocytes were wider but shorter, manifesting a rounded shape (Figure 5, B–D). The cardiomyocyte area was significantly enlarged, and the cardiomyocyte density was lower, suggesting cardiomyocyte hypertrophy (Figure 5, E–G). We also noticed an increase in the size of cardiomyocyte nuclei. Injection of an MMEJ sgRNA against *mtor* effectively rescued the size of cardiomyocyte nuclei but did not affect cardiomyocyte morphology, size, or density (Figure 5, B–G). In contrast, injection of an MMEJ sgRNA against *mapk3* specifically restored the prolonged cardiomyocyte shape by increasing the length and decreasing the width. The cardiomyocyte area, density, and nuclei size were not affected (Figure 5, B–G). Together, these data suggested distinct mechanisms underlying the therapeutic effects of *mtor* and *mapk3* inhibition on the VEC model (Figure 5H).

### Therapeutic effects of mTOR or MAPK inhibition are conserved in the VAC model.

**Figure 6:**
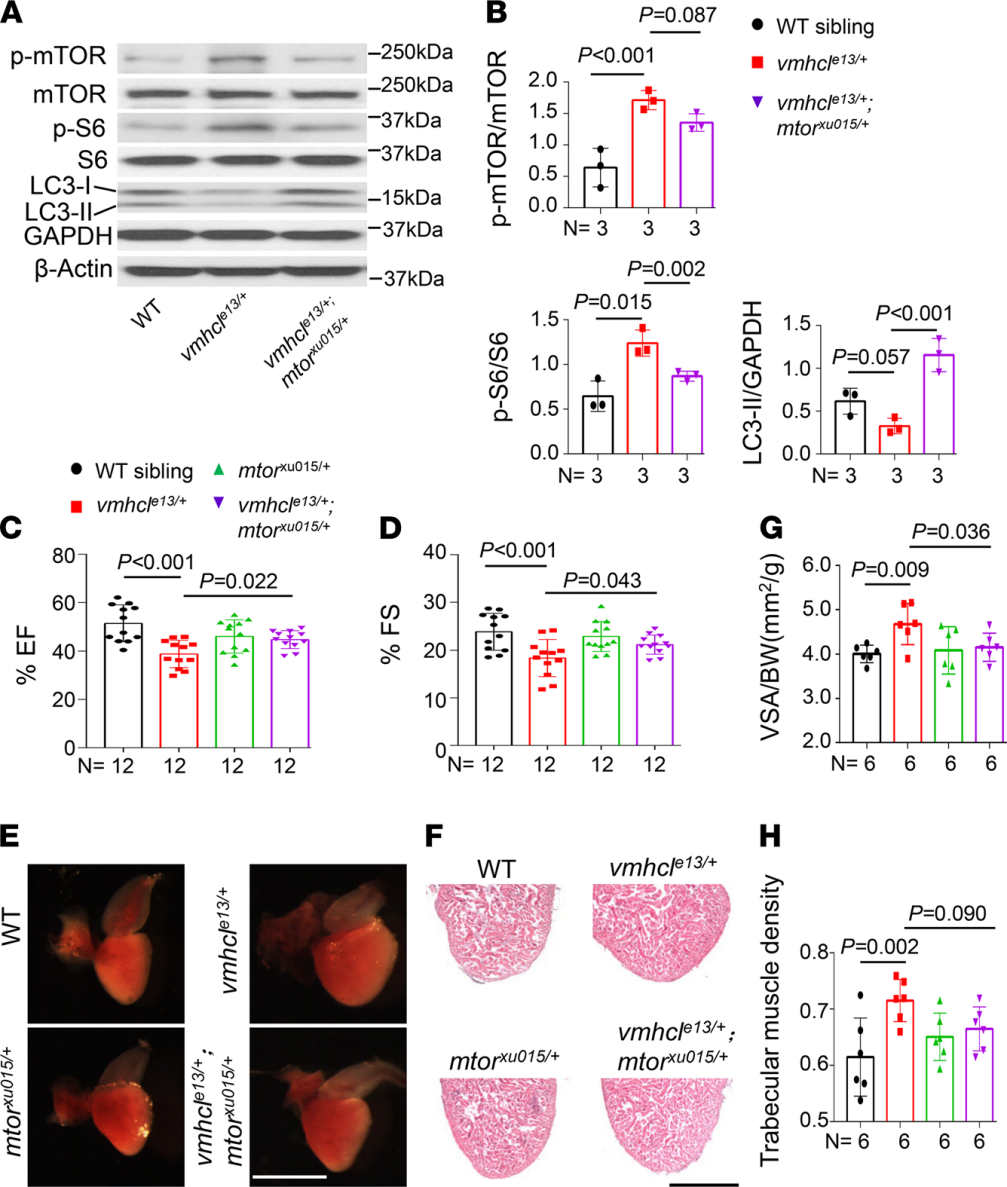
Therapeutic effects of *mtor* inhibition on the VAC model. (**A** and **B**) Representative Western blots showing the levels of proteins involved in mTOR signaling pathways in WT controls, *vmhcl^e13/+^*, and *vmhcl^e13/+^mtor^xu015/+^* mutants and quantification analysis. *n* = 3 biological replicates; data are presented as the mean ± SD; 1-way ANOVA. (**C** and **D**) Quantification of the percent EF and FS using echocardiography in fish at 8 months. *n* = 12; data are presented as the mean ± SD; 1-way ANOVA. (**E** and **F**) Representative images of isolated hearts and H&E staining in the apex area in fish at 8 months. (**G** and **H**) Quantification of the VSA normalized to BW and trabecular muscle density in these mutants and WT controls at 8 months. *n* = 6; data are presented as the mean ± SD; 1-way ANOVA. Scale bars: 2 mm in **E** and 300 μm in **F**.

Lastly, we tested whether CM signaling pathways identified in the VEC model were conserved in the VAC model. At the molecular level, we detected activated mTOR signaling, as indicated by hyperphosphorylation of mTOR, activation of pS6, and reduced levels of the LC3-II protein in the *vmhcl^e13/+^* mutants (Figure 6, A and B). In the *vmhcl^e13/+^mtor^xu015/+^* double mutants, *mtor^xu015+/−^* effectively reverted these molecular changes (Figure 6, A and B). Consequently, *mtor* inhibition significantly rescued the reduced cardiac function and enlarged heart size; nonetheless, the increased trabecular muscle density was not rescued (Figure 6, C–H). Similarly, we also noted partially restored cardiac function in the adult *vmhcl^e13/+^mapk3^MJ-F0^* double mutants (Supplemental Figure 10, A and B). Interestingly, we were able to identify 2 *vmhcl^e13/e13^mapk3^MJ-F1^* homozygous fish that survived to adulthood after genotyping approximately 200 adult fish generated from the *vmhcl^e13/+^ and mapk3^MJ-F1^* incrossing. Despite the low number of adult survivors (*n* = 2) and that 1 of the 2 *vmhcl^e13/e13^mapk3^MJ-F1^* homozygous fish displayed an enlarged ventricle and atrium (Supplemental Figure 10, C–E), this observation strongly suggests a rescuing effect of MAPK inhibition on the embryonic lethality of *vmhcl^e13/e13^* homozygosity. Taken together, these data confirmed the therapeutic effects of mTOR or MAPK inhibition on the zebrafish VAC model.

## Discussion

### vmhcl depletion and haploinsufficiency lead to CM-like phenotypes in embryonic and adult zebrafish, respectively.

In this study, through generating and characterizing the *vmhcl* mutants, we presented zebrafish as a useful vertebrate model for deciphering *MYH7* CM. The *vmhcl^e13/+^* mutant fish developed several characteristics of CM at 8 months of age, including reduced cardiac pump function, increased density of the trabecular muscle, and reduced exercise capacity. Because sarcomeric defects have yet to be detected (data not shown), comprehensive cardiac phenotyping analyses of *vmhcl^e13/+^* heterozygous mutant fish at ages beyond 8 months are needed in the future. At the genome level, the *vmhcl^e13/+^* mutant contains a small deletion in the exon 2 that shifts the reading frame and presumably results in early translational stop. At the mRNA level, we detected reduced levels of the *vmhcl* transcript and compensatory expression of other *myh* homologs, particularly *vmhc* and *amhc*. At the protein level, we noted reduced expression of overall Myh in the heart. Although we cannot eliminate the possibility of dominant negative effects incurred by a potentially truncated Vmhcl protein, our data strongly suggested that *vmhcl* loss-of-function is sufficient to trigger CM-like responses in this animal.

An important discovery of the present work is to demonstrate that the efficient VEC model can be used for deciphering CM signaling pathways. An assessment of 7 CM pathways via compound administration revealed 3 positive hits, 2 of which were subsequently validated by an independent genetic assessment of 11 target genes in all 7 signaling pathways. The therapeutic effects of both mTOR and MAPK inhibition were later confirmed in the VAC model. In fact, the possibility that zebrafish embryos can be used for deciphering the relationship between heart morphometry and cardiac function has been previously suggested by genetic studies of 2 other *myh* homologues, *vmhc* and *amhc* (47, 48). Unlike *vmhcl*, which is a predominant *myh* isoform in the adult ventricle, *vmhc* is the predominant *myh* isoform in the embryonic ventricle, and *amhc* is the predominant *myh* isoform in the atrium (40, 47, 48). Similar to *vmhcl^e13/e13^*, *haf,* a mutant affecting *vmhc*, displayed dramatically reduced ventricular contraction resulting in an enlarged ventricular chamber and enlarged cardiomyocytes (47). On the other hand, *wea*, a zebrafish mutant affecting *amhc*, has exhibited ablated atrial contractility resulting in a smaller ventricular chamber (49). The difference in the affected ventricular chamber size has been suggested to be a consequence of disrupted cardiac function in the ventricle and atrium, because the *haf;wea* double mutant rescued the increased ventricular size and the enlarged cardiomyocyte size (49). Together, our data strongly suggested that insights for *MYH7* CM could be gleaned by studying *myh* mutants in zebrafish embryos.

### mtor and mapk3 are 2 candidate therapeutic target genes for vmhcl-associated CM in zebrafish with different mechanisms.

Based on both the assessment of 7 CM pathway inhibitors and genetic testing of 11 candidate genes, we identified *mtor* inhibition as an approach to ameliorate VEC. We noted activated mTOR signaling in the VAC models and confirmed the therapeutic effects of *mtor* inhibition. Therapeutic effects similar to *mtor* inhibition have been observed on several other CM models, including anemia-induced CM (16), anthracycline-induced cardiotoxicity (16), *bag3*-associated DCM (14), and *lamp2*-associated HCM (13), suggesting that *mtor* signaling is a common pathological event in these CMs with distinct etiologies. Because aberrant protein quality control (PQC) is a common pathological event in many different CM types (50), it is possible that *mTOR* inhibition exerts its therapeutic effects via modulating PQC within cardiomyocytes. Intriguingly, we noted enlarged cardiomyocyte nuclei in the VEC model, which was rescued by mTOR inhibition but not MAPK inhibition. We postulated that this change in nuclear size is a consequence of defective mTOR signaling and/or PQC.

In addition to mTOR signaling, both our compound-based and genetic tests also converge on a therapeutic function of MAPK signaling inhibition in the VEC model. Because *ERK1/2* signaling was shown to be an important pathway that regulates the balance between eccentric and concentric growth of the heart (18), we propose that the *ERK1/2* signaling pathway could be the key hypertrophic growth pathway that confers aberrant cardiac function to regulate the shape of cardiomyocytes.

### Zebrafish are a valuable alternative animal model for MYH7 CM.

Besides the well-established advantage of enabling larger-scale compound screening in zebrafish (36–38, 51), our study highlighted the feasibility of rapidly assessing modifier genes in F0 fish using the MMEJ-based CRISPR/Cas9 genome editing technology. Effective gene knockdown with predictable genetic lesions can be reliably achieved in F0. Results from the transient genetic analysis in F0 could then be validated in the F1 generation. Because the technology eliminates the need for genetic crosses of multiple generations that are typically required for genetic interaction studies, unprecedently high throughput can be achieved for assessing modifier genes and therapeutic strategies.

In summary, this study established zebrafish as a vertebrate model for studying *MYH7* cardiomyopathy. Because of the conservation of the Myh6/Myh7 biology among zebrafish, larger mammals, and humans, but not rodents, our zebrafish VEC/VAC models possess great potential to serve as an alternative animal model to rodents. If inconsistent conclusions with rodents are noted, additional evidence from an *MYH7* CM model in larger mammals, such as a rabbit model (22), is recommended. Therefore, we anticipate that the integration of zebrafish as an animal model for *MYH7* CM will significantly accelerate mechanistic studies and therapeutic development.

## Methods

Supplemental Methods are available online with this article. All supporting data and materials described within the article will be made available upon reasonable request.

### Statistics.

The unpaired 2-tailed Student’s *t* test was used to compare data between 2 groups. To assess differences among multiple groups, 1-way or 2-way ANOVA was used, as appropriate. The log-rank test was used to determine the difference in animal survival. All quantitative data are presented as the mean ± SD. The sample size (*n*) represents the number of animals, unless otherwise specifically designated as the number of biological replicates. *P* values of less than 0.05 were considered significant. All statistical analyses were performed using GraphPad Prism.

### Study approval.

Zebrafish (*Danio rerio*; WIK strain) were maintained on a 14-hour light/10-hour dark cycle at 28.5°C. All animal study procedures were approved by Mayo Clinic Institutional Animal Care and Use Committee (protocol number: A3513) and performed in accordance with the Guide for the Care and Use of Laboratory Animals published by the US NIH (NIH publication no. 85-23, revised 1996).

## Author contributions

HB, YD, and XX conceptualized the project. HB, YD, JL, YHS, MW, and PZ performed experiments and analyzed and interpreted data. YZ analyzed the statistics. HB, YD, XL, and XX wrote the manuscript. All authors reviewed the manuscript and discussed the work

## Supplementary Material

Supplemental data

## Figures and Tables

**Table 2:**
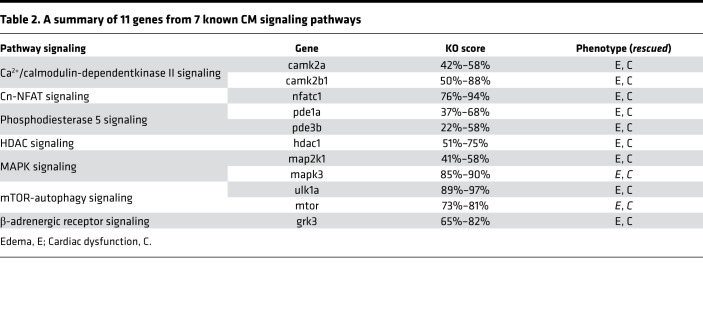
A summary of 11 genes from 7 known CM signaling pathways

**Table 1:**
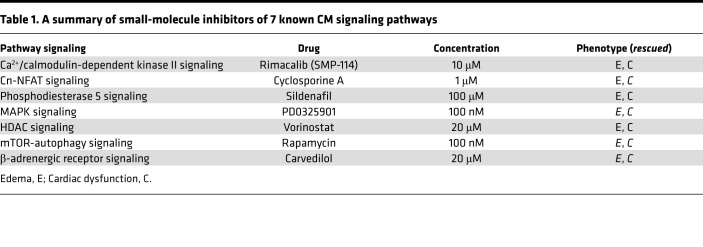
A summary of small-molecule inhibitors of 7 known CM signaling pathways

## Version 1 12/22/2021

Electronic publication
