# Supplementary material for: Inhibition of mTOR or MAPK ameliorates vmhcl/myh7 cardiomyopathy in zebrafish
Source: JCI Insight. 2021 Dec 22;6(24):e154215. doi: 10.1172/jci.insight.154215 (PMC8783688; doi:10.1172/jci.insight.154215)
Supplement: Supplemental data [file jciinsight-6-154215-s135.pdf]

Supplemental methods

Generation of vmhcl mutants. Transcription activator-like effector nuclease (TALEN) primer pairs targeting the 13th exon of the *vmhcl* gene were designed using Zifit (http://zifit.partners.org/ZiFiT/ChoiceMenu.aspx). Both TALENs were assembled using a Golden Gate kit (Addgene) (1). Capped mRNAs were synthesized using an mMESSAGE mMachine T3 Kit (Ambion). Approximately 25 pg of capped mRNAs were injected into one-cell-stage embryos. Founder fish (F0) were raised to adulthood and outcrossed to generate F1 embryos. Individual F1 embryos were used for genotyping with polymerase chain reaction (PCR) to identify mutant alleles (forward primer: 5′-GCTGAAGCTGATGGAACTG-3′; reverse primer: 5′-TGAAATACTGGCGAGGCTG-3′). The resulting PCR products were digested with the restriction enzyme HinfI to identify the WT or mutant genotype. The uncut PCR products were Sanger sequenced to determine the precise genomic lesions. Two different *vmhcl* mutant alleles that presumably resulted in different shifts of the reading frame for each mutant locus were selected for continuous outcrosses for 3 generations and then subjected to phenotypic analysis.

Quantification of cardiac function via video imaging at the embryonic stage. Zebrafish in the embryonic stage (days 3 to 4) were anesthetized with tricaine (0.02%) (Argent Chemical Laboratories) for 2 min, placed lateral side up, and held in place with 3% methylcellulose (Sigma-Aldrich). The beating hearts were documented by using a Zeiss Axioplan 2 microscope with differential interference camera (DIC) lens at the 10.0X magnification, and video clips were then used to calculate the heart rates and fraction shortening (FS) using the formula [FS=(Ld−Ls)/Ld]. FS was analyzed using ImageJ software. Ld and Ls represent the length of the short axis of the ventricle at the end-diastolic stage and end-systolic stage, respectively. The ventricular chamber volume (VCV) was calculated by the formula: volume = $(4/3)\ \pi ab^2$ (where a = long axis length and b = short axis length between the myocardial borders of ventricles).

Quantification of cardiac function using echocardiography at the adult stage. Cardiac function in the VAC model was measured and analyzed using a Vevo 3100 imaging system equipped with a 50 MHz linear array transducer (Fujifilm VisualSonics). Acoustic gel (Aquasonic® 100, Parker Laboratories) was applied over the surface of the transducer to provide adequate coupling with the tissue interface. Adult zebrafish were anesthetized with tricaine (0.02%) for 5 min, placed ventral side up, and held in place with a soft sponge stage. The 50 MHz (MX700) transducer was placed above the zebrafish to capture images of the sagittal imaging plane of the heart. B-mode images were acquired with a transmit focus at the center of the heart. Image

quantification was performed using the VevoLAB workstation. Data were acquired and processed as described in a recent report.(2) Cardiac contractility was quantified by calculating the ejection fraction (EF) [EF=(EDV−ESV)/EDV] or fractional shortening (FS) [FS=(Ld−Ls)/Ld], where EDV and ESV are the ventricular volumes at the end-diastolic stage and end-systolic stage, respectively. Ventricular chamber dimensions were measured from B-mode images using the following two indices: EDV/body weight (BW) and ESV/BW (3).

Swimming tunnel assay. The initial swimming capacity of *vmhcl* mutants was analyzed using our reported technique (4). Briefly, the swimming tunnel assay was conducted using a swim tunnel respirometer (Mini Swim 170, Loligo Systems). This protocol was derived from previous reports with modifications (5, 6). The *vmhcl* mutants were raised together with age-matched WT controls. All fish fasted for 24 h before the first swimming capacity measurement. To evaluate swimming capacity, nine adult fish in each group were transferred into the swim tunnel respirometer with an initial water speed of 9 cm/s for a 20-min acclimation period. Water flow was then increased in stages at a rate of 8.66 cm/s (Ti) every 150 s (Tii) until all fish were exhausted. The speeds were recorded at the last stage (Uii) and the previous stage (Ui) for each individual fish. The critical swimming capacity (Ucrit) was calculated with the following formula: Ucrit=Ui+[Uii×(Ti/Tii)] (4). Ucrit was then normalized to the body length (BL) of the corresponding individual. The same batches of fish were tested 48 h and 96 h later for validation.

Measurement of the ventricular surface area to body weight ratio. Owing to the small size of an adult zebrafish heart, we previously calculated the ventricular surface area (VSA) normalized to the BW as an index to assess heart size in an adult zebrafish.(7) Hearts of individual zebrafish were dissected and imaged next to a millimeter ruler under a Leica MZ FLI III microscope to measure the VSA. The largest projection of a ventricle was outlined using ImageJ software. Fish were anesthetized with a tricaine (0.02%) solution for 3 min, semidried on a paper towel, and weighed on a scale to measure BW. VSA/BW was then determined by calculating the largest projection area of the ventricle (in mm^2) divided by the bodyweight (in g).

Anatomy and histological staining of hearts. All zebrafish heart tissues were harvested from adult fish at the designated stages under a Zeiss microscope after euthanized by incubation with 0.05% tricaine for 10 min. The dissected tissues were immediately fixed with 4% PBS-buffered formaldehyde and sent to the Mayo Clinic Histology Core Laboratory for subsequent sample processing and paraffin embedding. All histology experiments were performed using paraffin sections cut on a microtome (Leica) that had been dried overnight at 37°C. Sections

were deparaffinized in xylene, rehydrated, and washed with distilled water. Then, the sections were subjected to hematoxylin and eosin (H&E) staining. The density of the trabecular muscle was quantified using ImageJ software.

Fluorescent antibody immunostaining. Fluorescent antibody immunostaining of zebrafish embryonic hearts was performed mostly as previously described (8, 9). Briefly, embryonic hearts at 3 days post-fertilization (dpf) were dissected using two BD U-100 insulin syringes and were transferred to slides with a 10 μl pipette. Dissected heart tissues were immediately fixed with 4% paraformaldehyde in PBS for 20 min at room temperature (RT), permeabilized with 0.1% Triton X-100 in PBS (PBST), blocked with 10% normal sheep serum in PBST for 1 h, and incubated with primary antibodies, including anti-troponin T (1:200, Sigma-Aldrich, T6277), anti-myosin heavy chain 1 (F59, 1:50; Developmental Studies Hybridoma Bank (DSHB)), anti-MEF2 (1:200, Abcam, ab197070), and anti-β-Catenin (Sigma-Aldrich, C7028) at 4°C overnight. After the hearts were washed with PBST three times for 10 min each, they were incubated for 1 h with secondary antibodies conjugated to Alexa Fluor 488 or 568 (1:200; Invitrogen). After the heart tissues were washed with PBST for 10 min three times, they were incubated with the mounting medium containing DAPI (Vector Laboratories). After image acquisition, the genotypes of all embryos used were determined by PCR and Sanger sequencing. The hearts were then imaged and analyzed using a Zeiss Axioplan 2 microscope equipped with Apotome (Carl Zeiss).

Quantitative RT-PCR. Whole embryos at 3 dpf and heart tissues from adult zebrafish were harvested after euthanization by incubation with 0.032% tricaine for 10 min. Tissues were homogenized using a Bullet Blender tissue homogenizer (Next Advance Inc.), and total RNA was extracted using TRIzol (Sigma) according to the manufacturer's instructions. 500 ng Isolated total RNA was used to generate cDNAs using the Superscript III First-Strand Synthesis System (Invitrogen). Quantitative RT-PCR was carried out in 96-well qPCR plates (Applied Biosystems). Gene expression was normalized to the expression level of *gapdh* using –ΔΔCt (cycle threshold) values. The primers used were 5'-GCGATGCTGAAATGTCTGTT-3' (sense) and 5'-CAGTCACAGTCTTGCCTCCT-3' (antisense) for *vmhcl*, 5'-GCAGGAATACACAATCCGC-3' (sense) and 5'-GCTTCCTTTACAGTTACAGTCTTTC-3' (antisense) for *vmhc*, 5'-TCAGATGGCAGAGTTTGGAG-3' (sense) and 5'-CGTGTATCTGGAGTGAAACTG-3' (antisense) for *nppa*, 5'-GCAGGAATACACAATCCGC-3' (sense) and 5'- CGTGTATCTGGAGTGAAACTG-3' (antisense) for *nppb*, 5'-TGAAGACCTGAGAAGGCAAC-3' (sense) and 5'-CAGTTCCTCGGTTCTCTGAA-3' (antisense)

for *myh6 (amhc)*, 5'-GCCTGAACTTCTTGACATGC-3' (sense) and 5'-CTCCCTCTGCTTCTGTTTGA-3' (antisense) for *myh7ba*, 5'-GAACTCCAAGACATGCTGCT-3' (sense) and 5'-TTGAACTTCATGTTGCCAAA-3' (antisense) for *myh7bb*, and 5'-CCACCCATGGAAAGTACAAG-3' (sense) and 5'-CTCTCTTTGCACCACCCTTA-3' (antisense) for *gapdh.*

Western blotting. Western blotting was performed as described previously (7). Hearts dissected from adult fish were transferred immediately to RIPA buffer (Sigma-Aldrich) supplemented with a protease inhibitor cocktail (Roche) and homogenized using a Bullet Blender tissue homogenizer (Next Advance). The resulting protein lysates were subjected to western blotting using a standard protocol. The following primary antibodies were used: anti-Gapdh (1:1000, Santa Cruz Biotechnology, sc-25778), anti-mTOR (Ser2448) (1:1000, Cell Signaling Technology, 2983), anti-phospho-mTOR (Ser2448) (1:3000, Cell Signaling Technology, 2971), anti-phospho-S6 ribosomal protein (Ser240/244) (1:5000, Cell Signaling Technology, 2215), anti-S6 ribosomal protein (1:8000, Cell Signaling Technology, 2217), anti-LC3 (1:3000, Cell Signaling Technology, 12741).

Coomassie blue staining. All zebrafish heart tissues were harvested from adult fish at the designated stages under a Zeiss microscope after euthanized by incubation with 0.032% tricaine for 10 min. Fresh hearts isolated from adult fish were transferred immediately to RIPA buffer (Sigma-Aldrich) supplemented with a complete protease inhibitor cocktail (Roche) and homogenized using a Bullet Blender tissue homogenizer (Next Advance). The resulting protein lysates were resolved on a mini-protein TGX precast gel using a standard protocol. After electrophoresis, the gel was transferred into a plastic or glass tray for staining. A sufficient amount of Coomassie brilliant blue R-250 staining solution (45 ml of methanol, 10 ml of acetic acid, 45 ml of ddH2O, and 0.25 g of Coomassie brilliant blue R-250) was added to cover the gel, allowing it to move freely in the tray overnight. The tray was covered with plastic wrap. Bands should be evident on the gel at this point. The staining solution was removed and then a sufficient amount of destaining solution was added (50 ml of methanol, 40 ml of acetic acid, and 10 ml of ddH2O) to cover the gel, allowing it to move freely in the tray for 4-6 h. The tray was covered with plastic wrap. After destaining, much of the background staining of the gel disappeared, and the stained protein bands should be clear. The destaining solution was removed and the gel was scanned and analyzed.

Assessment of small-molecule inhibitors. Fish embryos at 8 hours post-fertilization (hpf) were treated with small-molecule inhibitors for 48 h. The percentage of embryos with cardiac edema and cardiac function were measured using ImageJ software at the end of the treatment. The following concentrations were used for each small-molecule inhibitor: Rimacalib (10 μM, MedChemExpress, HY-100779), Cyclosporine A (1 μM, Apex Bio, B1922), Sildenafil (100 μM, Sigma-Aldrich, PZ0003), PD0325901 (100 nM, Sigma-Aldrich, PZ0162), Vorinostat (20 μM, Sigma-Aldrich, SML0061), Rapamycin (100 nM, Sigma-Aldrich, R0395), and Carvedilol (20 μM, Sigma-Aldrich, C3993).

Assessment of genetic modifying effects on F0 fish using MMEJ-based genome editing technology. Eleven genes from 7 known signaling pathways were experimentally tested using MMEJ technology (10-12). Sequences of these 11 genes were acquired from Ensemble (https://uswest.ensembl.org/index.html). The sequences of exons were then uploaded to an online algorithm, MENTHU (http://genesculpt.org/menthu/). High score target sequences were chosen from predicted candidate MMEJ loci. Guide RNAs (crRNA+ tracrRNA) with modifications were obtained from Synthego (https://www.synthego.com/). Guide RNAs (100 μM) were dissolved in 15 μl of nuclease-free duplex buffer (Integrated DNA Technologies, 11-01-03-01) upon arrival and then diluted to the different target concentrations (1 μM, 5 μM, or 10 μM) before use. The Alt-R Cas9 protein (Integrated DNA Technologies, 1081058) was diluted in working buffer (20 mM HEPES, 100 mM NaCl, 5 mM $MgCl_2$, 0.1 mM EDTA at pH 7.5) to the target concentration (3.3 μg/μl). Guide RNAs and the Cas9 protein were mixed 1:1 (1.5 μl of gRNA+1.5 μl of Cas9 protein) and incubated in a 37°C water bath for 10 min to assemble the Cas9 protein-gRNA complex. Then, the complex was injected into embryos. Embryos were sacrificed for DNA extraction that was used as a template for PCR to quantify the knockdown efficiency. The PCR amplicon was purified by ExoI digestion and subsequently sent for Sanger sequencing at Genewiz (https://clims4.genewiz.com/CustomerHome/Index). The chromatograms from both uninjected and injected amplicons were used for the analysis of knockout efficiency with the R code-based open-access software Inference of CRISPR Edits (ICE) v2 CRISPR Analysis Tool (https://www.synthego.com/products/bioinformatics/crispr-analysis). Sequences for guide RNAs and primers for these 11 genes are listed in Supplemental Table 1. After image acquisition, the genotypes of all embryos were determined by PCR and Sanger sequencing. Individual *mapk3*$^{e2\text{-}MJ}$ F1 embryos were used for genotyping with PCR to identify mutant alleles (forward primer: 5′-CGCACTGTGAGTGTTTAAGG-3′; reverse primer: 5′-AACTATTTCTTTCCCTGCTGC-3′). The resulting PCR products were digested with the

restriction enzyme DdeI to identify the WT or mutant genotype. The uncut PCR products were Sanger sequenced to determine the precise genomic lesions. The *vmhcl*$^{e13/e13}$, wild-type, and double mutant fish were selected for cardiac function analysis.

Measurements of the cardiomyocyte size, area, and density. Fluorescent immunostaining using an anti-β-Catenin antibody (1:200, Sigma, C7082) for the cell membrane and an anti-myocyte enhancer factor-2 (anti-MEF2) antibody (1:200, Abcam, ab197070) for cardiomyocyte (CM) nuclei was performed in isolated embryonic zebrafish hearts according to a protocol described previously to measure the CM size and nuclei (9, 13). A Zeiss Axioplan 2 microscope was used for imaging. Six cells in the outer curvature region (OCR) of each individual heart were selected for measuring the cell surface area using ImageJ software (13, 14). Only CMs with clear outlines were chosen for the measurement. Measurements from six fishes per group were obtained to calculate the average CM length, width, area, and nuclei size. For cell density, a square of at least 5,000 μm^2 per ventricle section per fish was analyzed using ImageJ software. Cells in that square were counted and divided by the square area to obtain cells per mm^2.

Supplemental References

1. Cermak T, Doyle EL, Christian M, Wang L, Zhang Y, Schmidt C, Baller JA, Somia NV, Bogdanove AJ, and Voytas DF. Efficient design and assembly of custom TALEN and other TAL effector-based constructs for DNA targeting. *Nucleic Acids Res.* 2011;39(12):e82.
2. Wang LW, Huttner IG, Santiago CF, Kesteven SH, Yu ZY, Feneley MP, and Fatkin D. Standardized echocardiographic assessment of cardiac function in normal adult zebrafish and heart disease models. *Dis Model Mech.* 2017;10(1):63-76.
3. Zhang H, Dvornikov AV, Huttner IG, Ma X, Santiago CF, Fatkin D, and Xu X. A Langendorff-like system to quantify cardiac pump function in adult zebrafish. *Dis Model Mech.* 2018;11(9).
4. Ding Y, Dvornikov AV, Ma X, Zhang H, Wang Y, Lowerison M, Packard RR, Wang L, Chen J, Zhang Y, et al. Haploinsufficiency of mechanistic target of rapamycin ameliorates bag3 cardiomyopathy in adult zebrafish. *Dis Model Mech.* 2019;12(10).
5. Sun Y, Fang Y, Xu X, Lu G, and Chen Z. Evidence of an Association between Age-Related Functional Modifications and Pathophysiological Changes in Zebrafish Heart. *Gerontology.* 2015;61(5):435-47.
6. Wang J, Panakova D, Kikuchi K, Holdway JE, Gemberling M, Burris JS, Singh SP, Dickson AL, Lin YF, Sabeh MK, et al. The regenerative capacity of zebrafish reverses cardiac failure caused by genetic cardiomyocyte depletion. *Development.* 2011;138(16):3421-30.
7. Ding Y, Sun X, Huang W, Hoage T, Redfield M, Kushwaha S, Sivasubbu S, Lin X, Ekker S, and Xu X. Haploinsufficiency of target of rapamycin attenuates cardiomyopathies in adult zebrafish. *Circ Res.* 2011;109(6):658-69.
8. Shih YH, Dvornikov AV, Zhu P, Ma X, Kim M, Ding Y, and Xu X. Exon- and contraction-dependent functions of titin in sarcomere assembly. *Development.* 2016;143(24):4713-22.

9. Yang J, and Xu X. Immunostaining of dissected zebrafish embryonic heart. *J Vis Exp.* 201259):e3510.
10. Hoshijima K, Jurynec MJ, Klatt Shaw D, Jacobi AM, Behlke MA, and Grunwald DJ. Highly Efficient CRISPR-Cas9-Based Methods for Generating Deletion Mutations and F0 Embryos that Lack Gene Function in Zebrafish. *Dev Cell.* 2019;51(5):645-57 e4.
11. Wu RS, Lam, II, Clay H, Duong DN, Deo RC, and Coughlin SR. A Rapid Method for Directed Gene Knockout for Screening in G0 Zebrafish. *Dev Cell.* 2018;46(1):112-25 e4.
12. Shah AN, Davey CF, Whitebirch AC, Miller AC, and Moens CB. Rapid reverse genetic screening using CRISPR in zebrafish. *Nat Methods.* 2015;12(6):535-40.
13. Yang J, Hartjes KA, Nelson TJ, and Xu X. Cessation of contraction induces cardiomyocyte remodeling during zebrafish cardiogenesis. *Am J Physiol Heart Circ Physiol.* 2014;306(3):H382-95.
14. Auman HJ, Coleman H, Riley HE, Olale F, Tsai HJ, and Yelon D. Functional modulation of cardiac form through regionally confined cell shape changes. *PLoS Biol.* 2007;5(3):e53.

Supplemental Table 1: List of the sequences for MMEJ-inducing guide RNA and primers for quantifying knockout score

Target Genes	sgRNA Sequence	MENTHU Score	Frame Shift	Strand	Deletion Sequence	PCR Sequence
camk2a	GCTGTGAAGTTGGCAGATTTTGG	3.12	Yes	forward	AGATTTTGGC	F: GCCAATGTCTGGTGGTACAG<br>R: AAAATCTGAACACTGAAGGAGC
camk2b1	CAAGAACGCTGCTGTGAAGCTGG	1.16	Yes	forward	TGAAGCTG	F: AAATCATGTAGGAGCATCGC<br>R: TGTGTTTAAGGCAGGAGAAATG
nfatc1	ATTCTCCTTGTGGCGGTAAACGG	2.71	Yes	forward	TAAACGG	F: TGCTTCTCCACGCCATTCTC<br>R: AACCAACTGTCCTCCGTCAC
pde1a	CTCCACCTTTACCAGGAAGATGG	1.37	Yes	forward	AGATGGGA	F: AACCGCCACAAAAATGAATG<br>R: TGTGCTGTACACTCTGAGC
pde3b	CCACGTAGATAGCCGAGCCGCGG	2.08	Yes	complement	CTCGG	F: ACTGGGAGAAATCCAAGAGG<br>R: CTATGTGAAGAGCTGCGTC
hdac1	CCGCTGCTCATGCTGGGAGGTGG	1.42	Yes	forward	GAGGTGG	F: ATTTCTAAGTTGGCTAGGCTTC<br>R: AGAGAAAATCTTCCAAGGCAG
map2k1	CCCTACATCGTGGGCTTCTATGG	1.36	Yes	forward	CTATGGGGCTT	F: TTATGCAGTTGATCCACCTTG<br>R: AGCCCATTTAACAGTAGTGTG
mapk3	GGAGGATTTTGATCTCTCTCAGG	1.67	Yes	complement	AGAG	F: CGCACTGTGAGTGTTTAAGG<br>R: AACTATTTCTTTCCCTGCTGC
ulk1a	ATCCTTCTCTCATACAGCACAGG	3.18	Yes	forward	CACAG	F: CTGATCCATAACCAATCTGCG<br>R: GTCTGAGTGAGGACACCATC
mtor	GAAAGAGAAAGGGATGAATAAGG	1.58	Yes	forward	ATAAGGATGA	F: AGGTGCAGCCATTCTTTGAT<br>R: TCATACCTCTCCCTCCATAC
grk3	TGAGGTTCTGCAGAAGGGAACGG	1.40	Yes	forward	GGGAA	F: TTGAGCTGTTGTGTTTAGCG<br>R: ACACATCAAACACCGAGCG

MMEJ: microhomology-mediated end joining; PCR: polymerase chain reaction.

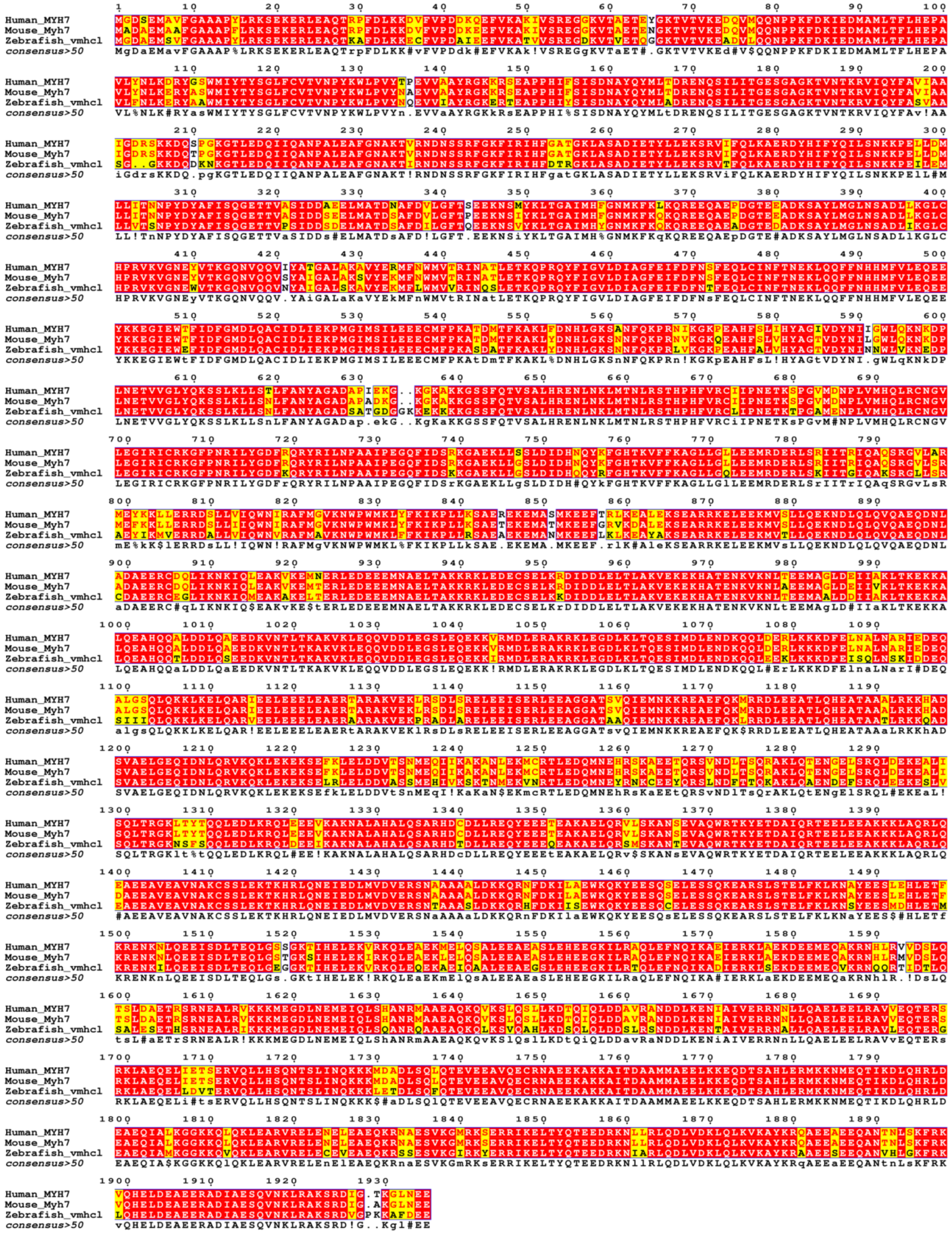

Supplemental Figure 1. Sequence alignment of human MYH7, mouse Myh7, and zebrafish Vmhcl proteins.

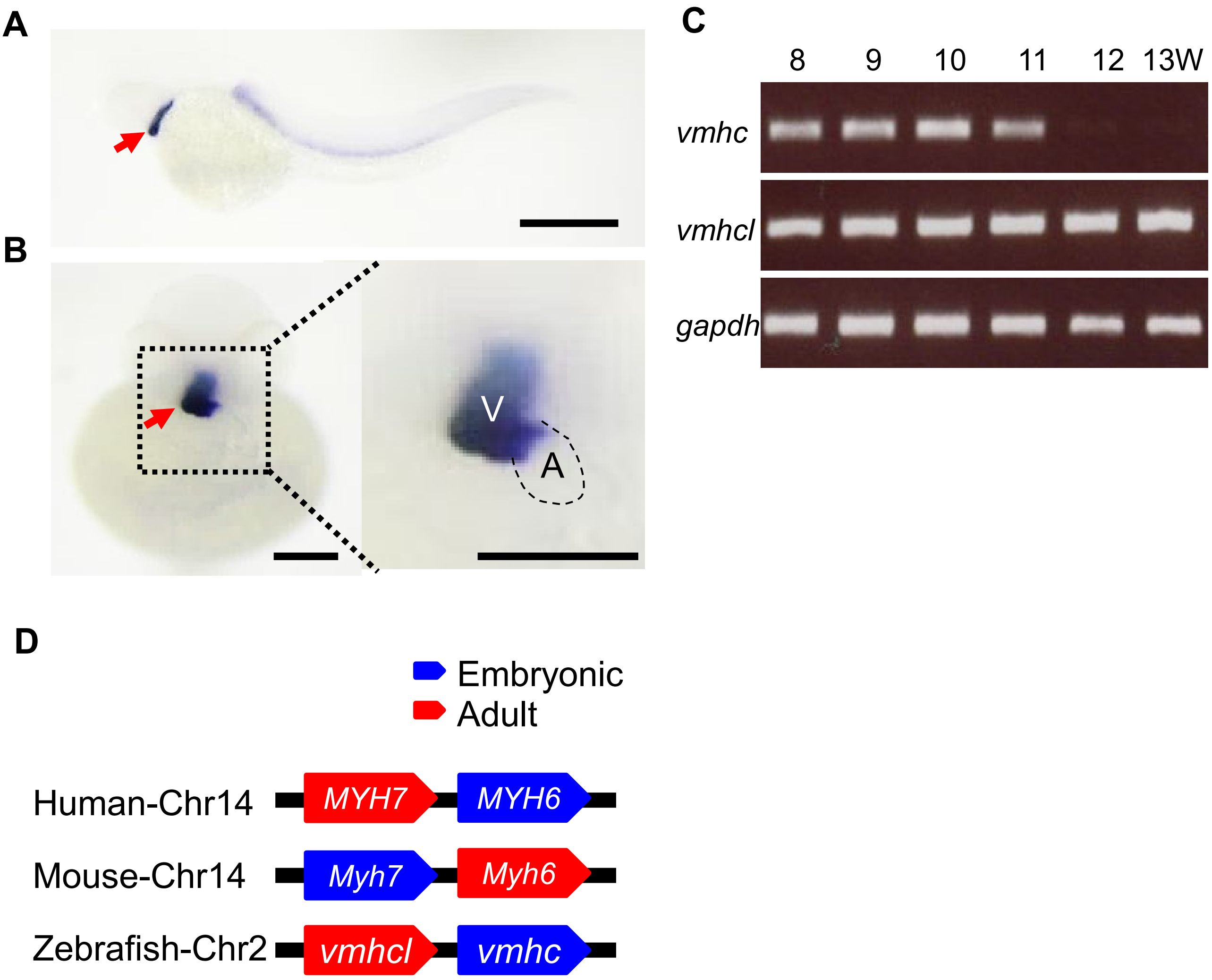

Supplemental Figure 2. Similar to human *MYH7/beta MYH*, *vmhcl* is a predominant *MYH* homolog expressed in the adult zebrafish ventricle. (A-B) Whole-mount in situ hybridization of 2 dpf embryos. *vmhcl* transcripts are mainly expressed in both embryonic hearts (red arrow) and somites. Within the heart, its expression is restricted to the ventricle, and it is not detected in the atrium. Scale bars: 500 μm. **(C)** Changes in the RNA expression level analyzed using semi-quantitative reverse transcriptase polymerase chain reaction (semi-qPCR) at different stages (weeks) of development. *gapdh* was used as a control. **(D)** Schematics of the genomic regions of *MYH6/MYH7* homologs in humans, mice, and zebrafish. Chr indicates chromosome.

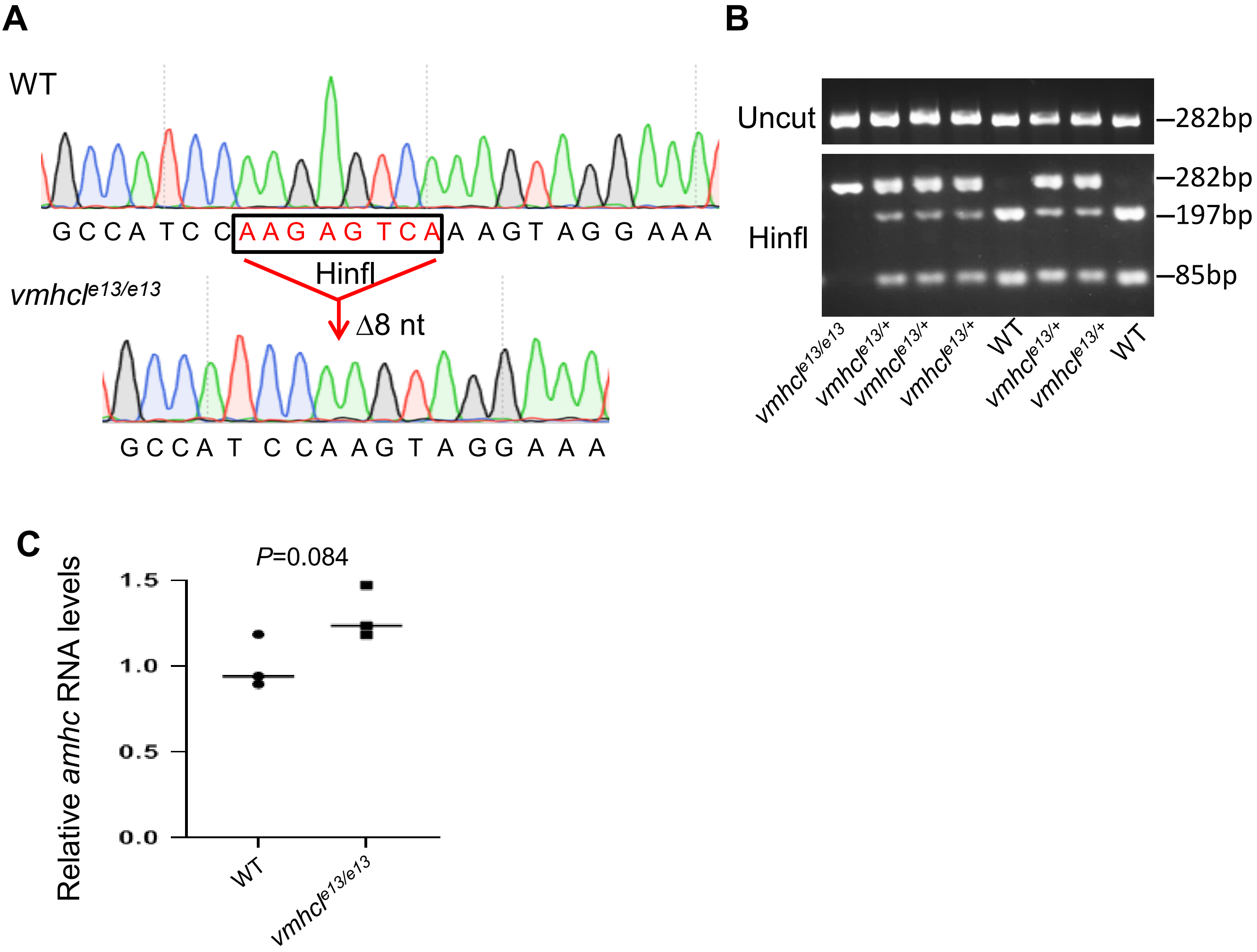

Supplemental Figure 3. Generation of a *vmhcl* mutant allele. (A) Chromographs illustrating the sequences of wild-type *vmhcl* and 8 nucleotide deletion mutant allele (*vmhcl*^{e13}). The boxed sequence indicates the region containing sites for the restriction enzyme HinfI in the WT sequence that was deleted in the mutants. **(B)** Representative agarose gel image showing the genotyping PCR results to identify the 8 nucleotide deletion allele and to differentiate among *vmhcl* heterozygous (*vmhcl*$^{e13/+}$), homozygous (*vmhcl*$^{e13/e13}$) mutants, and WT controls. **(C)** Quantitative RT-PCR analysis of *amhc (myh6)* gene transcript levels in *vmhcl*$^{e13/e13}$ mutant hearts compared to WT control at 3 days post-fertilization (dpf). n=3 biological replicates, Student's *t*-test.

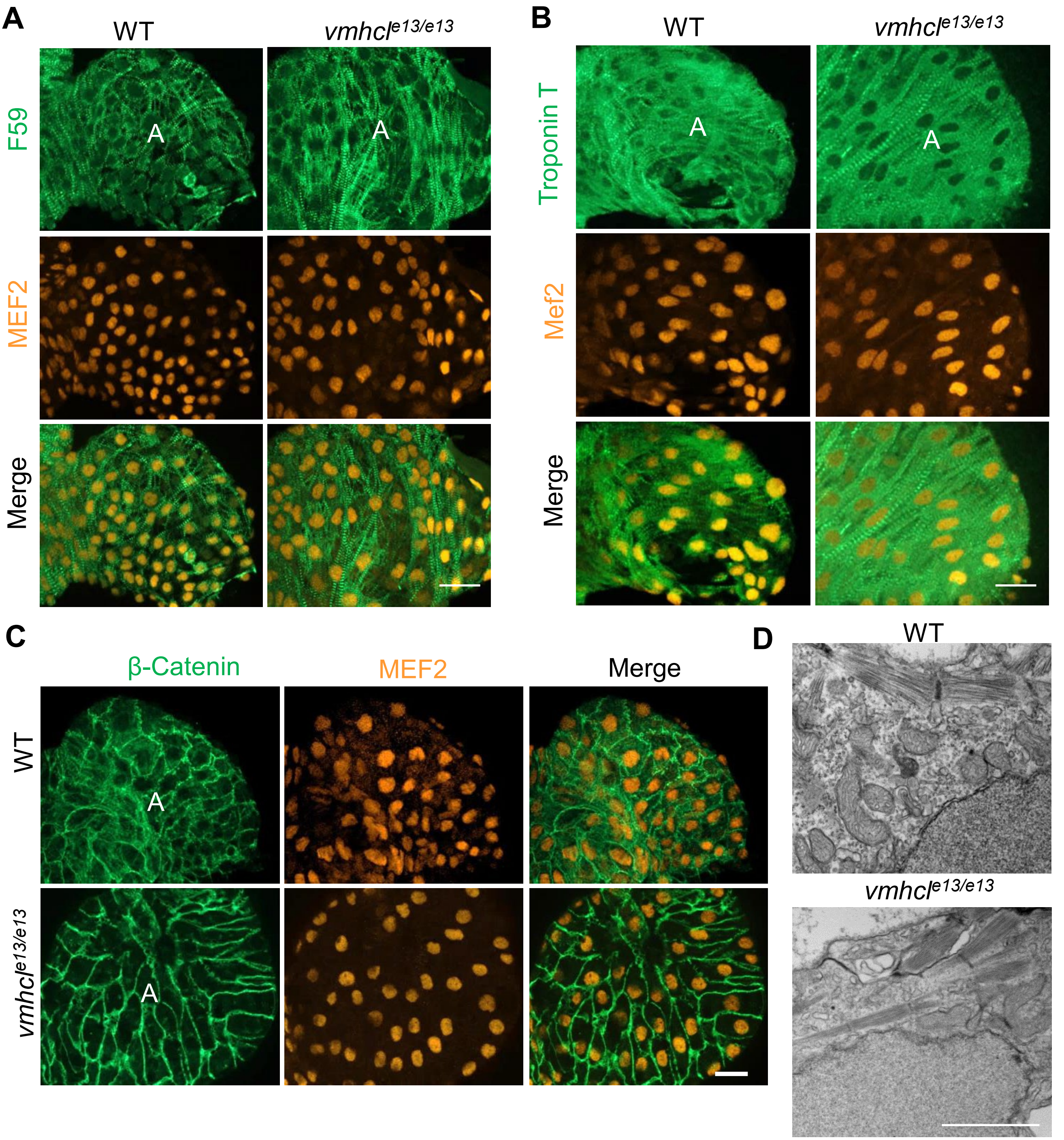

Supplemental Figure 4. *vmhcl* depletion does not affect sarcomere integrity in the atrium, but increases the cardiomyocyte cell size concurrent with reduced cardiomyocyte density. (A-B) Fluorescent co-immunostaining using anti-myosin heavy chain 1 (F59) (A) and anti-troponin T antibody (B) and anti-Mef2C antibody indicated the sarcomere structure in the *vmhcl*$^{e13/e13}$ mutant atrium is largely comparable to WT control at 3 dpf. dpf, days post fertilization. A, atrium. Scale bars: 20 μm. **(C)** Fluorescent Co-immunostaining using anti-β-catenin and anti-Mef2C antibody indicated the cardiomyocyte cell size appears to be enlarged concurrent with reduced cardiomyocyte density in the *vmhcl*$^{e13/e13}$ mutant atrium compared to WT control at 3 dpf. A, atrium. Scale bars: 20 μm. **(D)** Representative transmission electronic microscopy (TEM) images confirmed the preserved sarcomere structure in the atrium of *vmhcl*$^{e13/e13}$ mutant hearts. Scale bar: 2 μm.

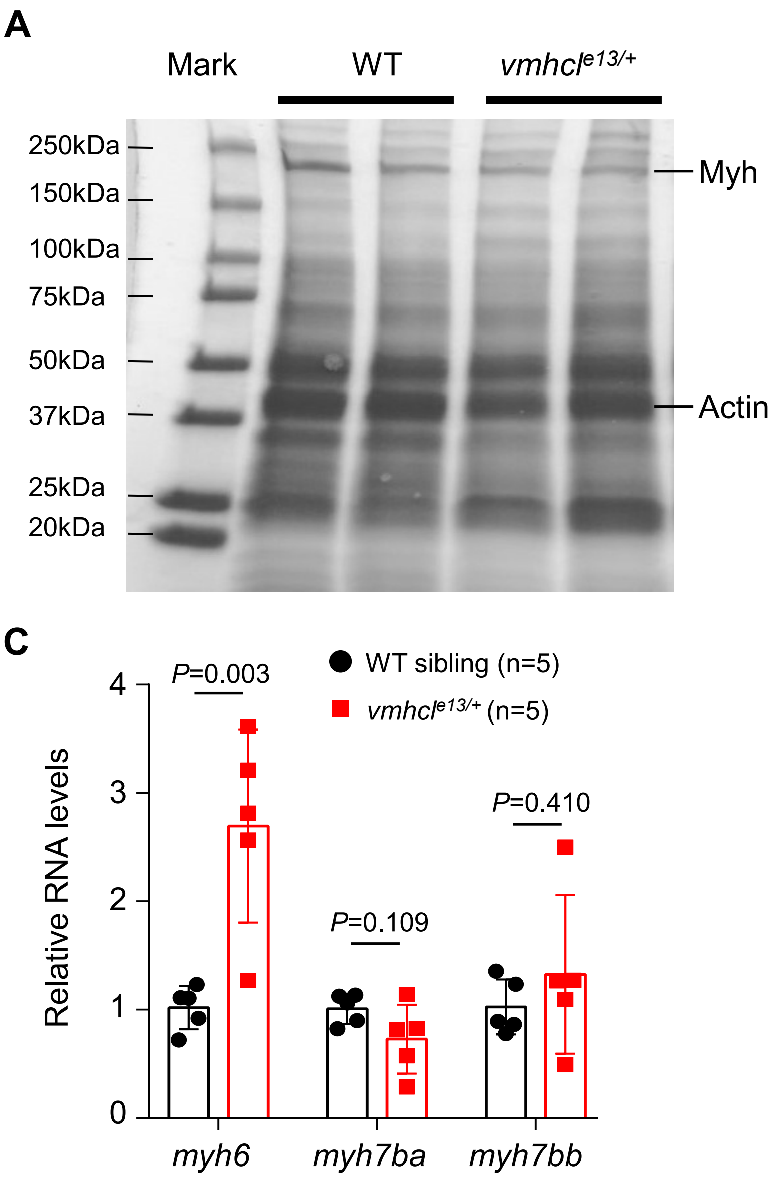

Supplemental Figure 5. Altered expression of *MYH* homologs at the protein and mRNA levels in adult *vmhcl*$^{e13/+}$ fish. **(A)** Myh protein expression in the hearts of adult fish was analyzed using Coomassie blue staining. **(B)** Quantification of Myh/Actin expression ratio in the *vmhcl*$^{e13/+}$ mutant and WT control at 9 months. n=4, Data are presented as the means±s.d. **(C)** Quantitative RT-PCR analysis of *MYH* homologs (*amhc*, *myh7ba*, and *myh7bb*) in *vmhcl*$^{e13/+}$ mutant hearts. n=5 biological replicates, Student's *t*-test

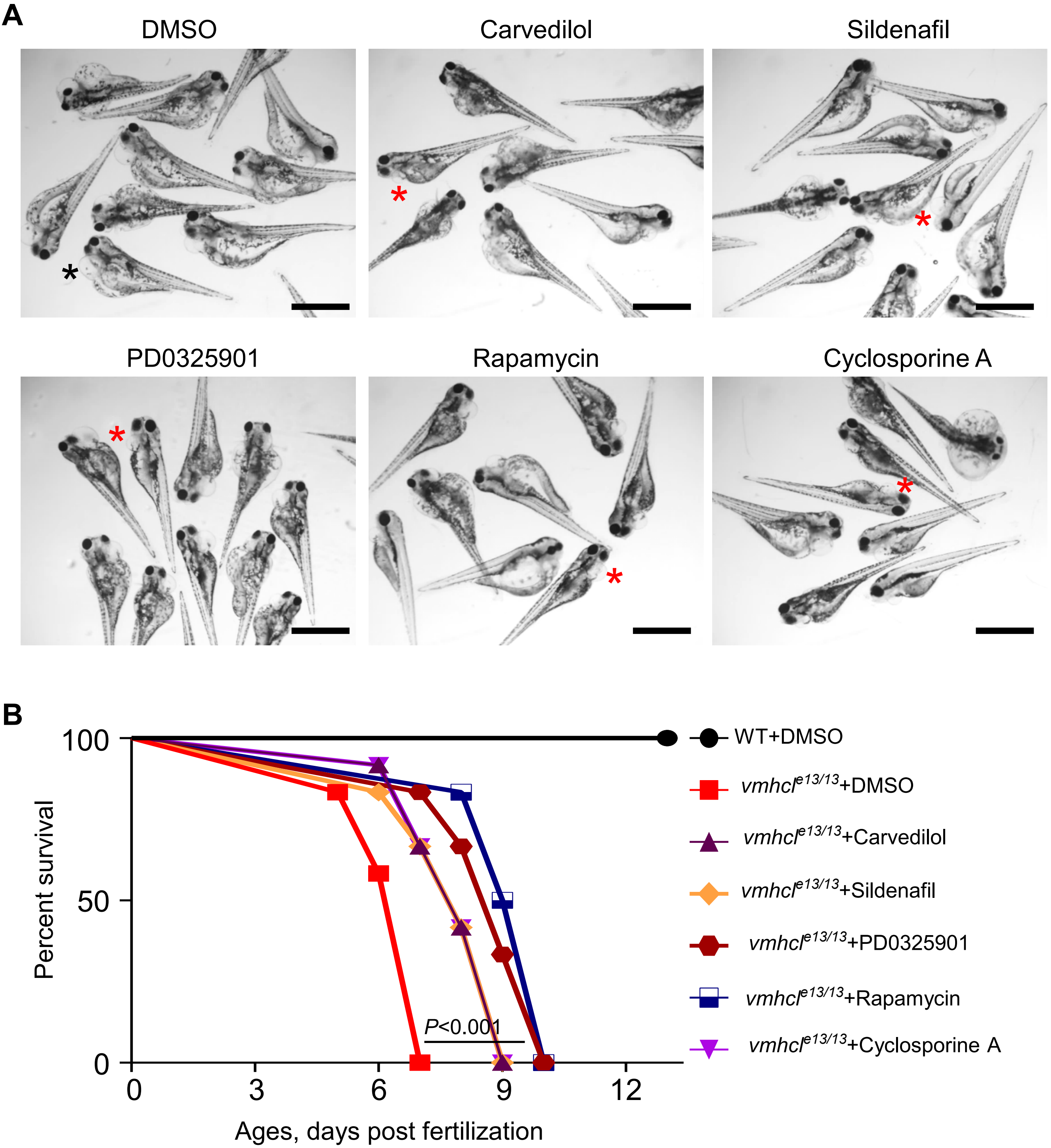

Supplemental Figure 6. Phenotypes of the VEC model treated with potential therapeutic compounds. **(A)** Images of *vmhcl*$^{e13/e13}$ mutant fish after drug administration compared with DMSO at 5 dpf. Black star indicates the severe pericardial edema phenotype. Red stars indicate milder edema. dpf, days post fertilization. Scale bar: 1 mm. **(B)** Kaplan–Meier survival curves of *vmhcl*$^{e13/e13}$ mutant fish treated with DMSO and drugs compared with WT controls. n=12, log-rank test.

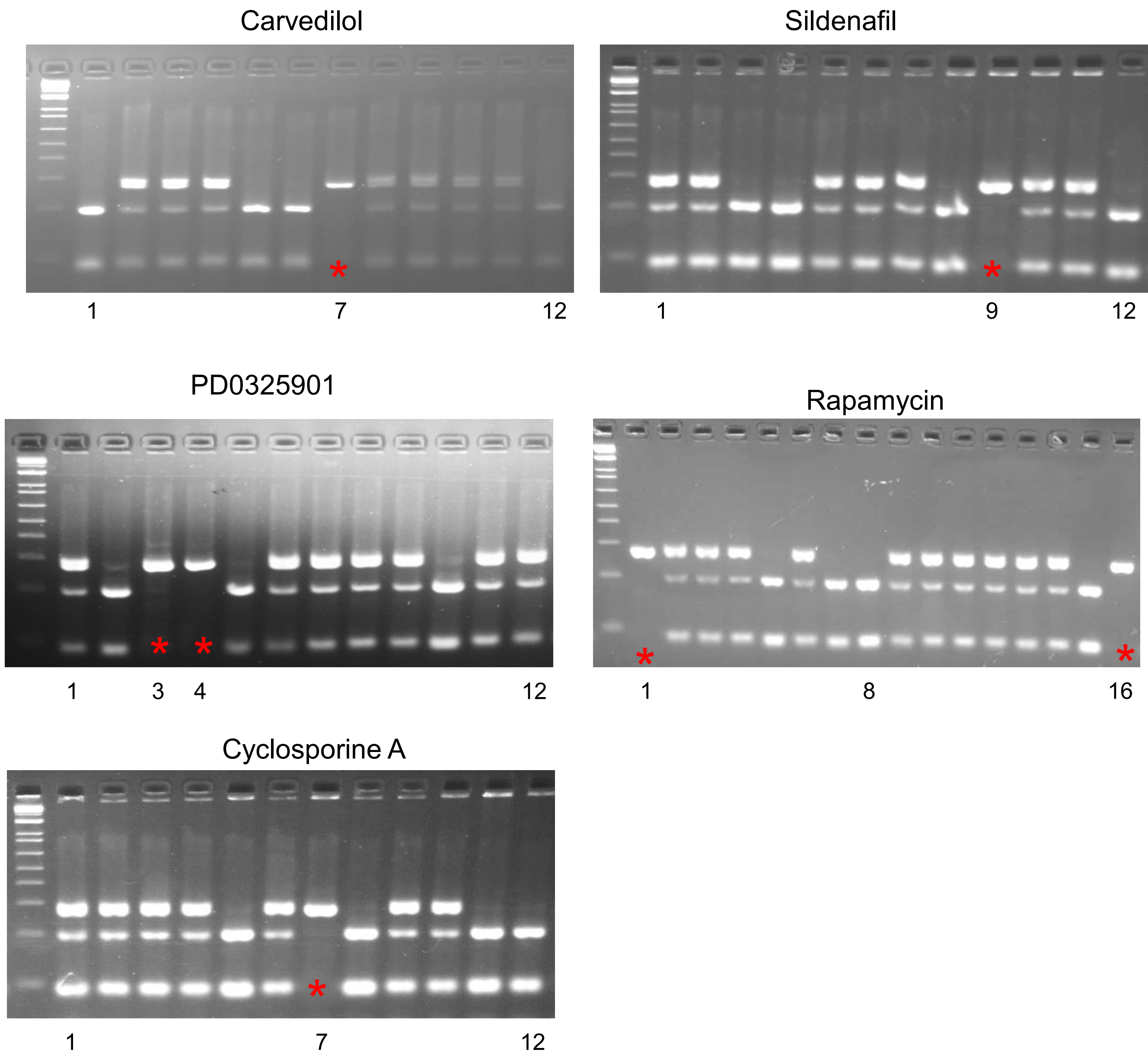

Supplemental Figure 7. Edema phenotypes in some *vmhcl*$^{e13/e13}$ fish can be rescued by administration of 5 different drugs. Shown are representative genotyping results of *vmhcl*$^{e13/e13}$ homozygous fish without obvious pericardial edema after drug treatment. Red stars indicate samples with the *vmhcl*$^{e13/e13}$ genotype.

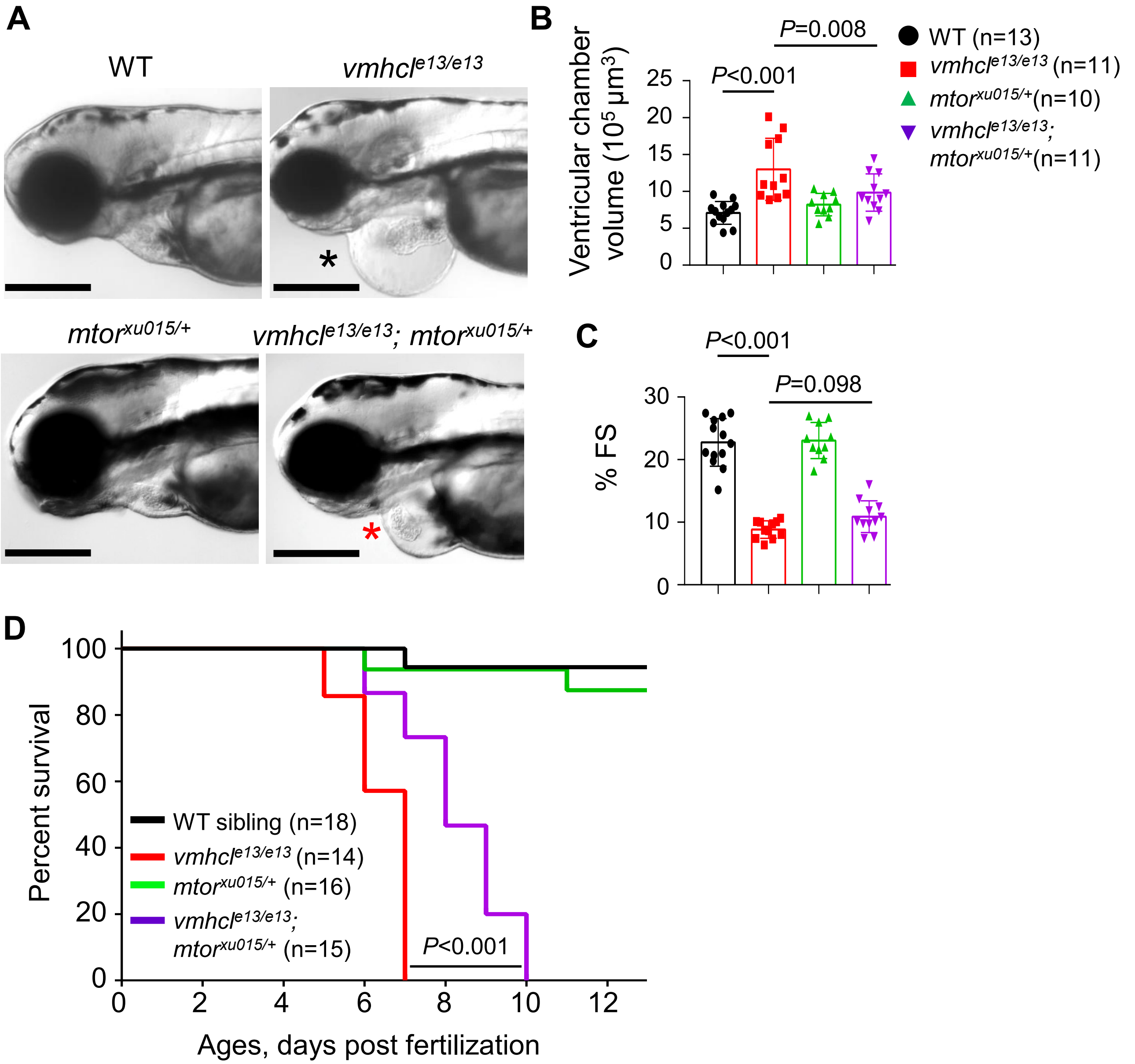

Supplemental Figure 8. Salutary modifier effects of MMEJ-based *mtor* inhibition are confirmed in the stable double mutants. (A) Representative images of the *vmhcl*$^{e13/e13}$, *mtor*$^{xu015/+}$ and *vmhcl*$^{e13/e13}$;*mtor*$^{xu015/+}$ mutant fish and WT controls at 5 dpf. The black star indicates severe edema. The red star indicates mild edema. dpf, days post fertilization. Scale bar: 300 μm. **(B-C)** Quantification of the ventricular chamber volume and percent fractional shortening (FS) of *vmhcl*$^{e13/e13}$, *mtor*$^{xu015/+}$, *vmhcl*$^{e13/e13}$;*mtor*$^{xu015/+}$ mutant fish compared with WT controls at 5 dpf. n=10-13, Data are presented as the means ± s.d. One-way ANOVA was used for statistical analyses. **(D)** Kaplan–Meier survival curves of *vmhcl*$^{e13/e13}$, *mtor*$^{xu015/+}$, *vmhcl*$^{e13/e13}$;*mtor*$^{xu015/+}$ mutant fish and WT controls. n=14-18, log-rank test.

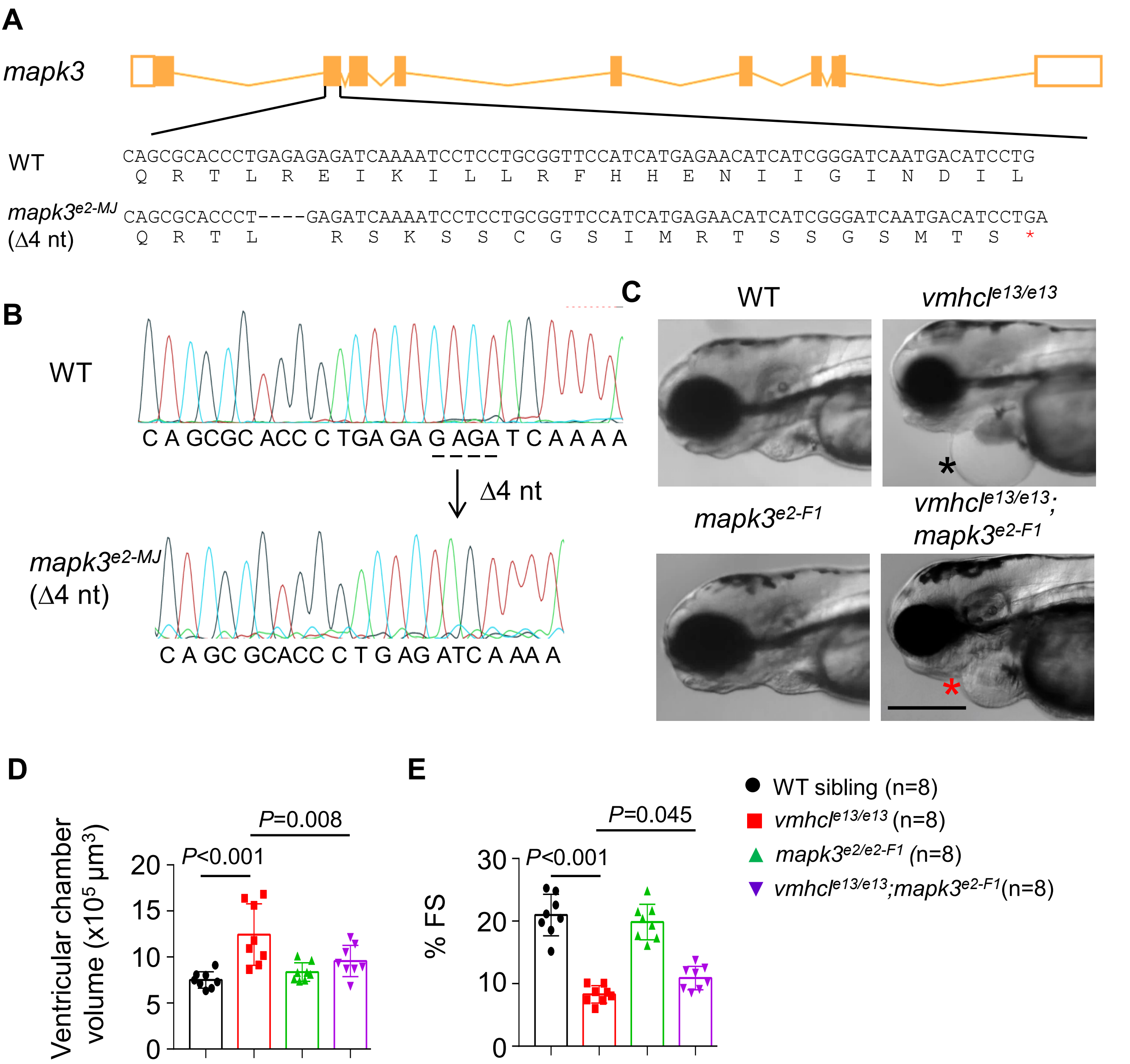

Supplemental Figure 9. Salutary modifying effects of MMEJ-based *mapk3* inhibition are confirmed in the stable double mutants. (A) Schematics of MMEJ-based *mapk3* genetic lesion of 4-nucleotides generated by injection of a sgRNA targeting on sequences in the 2nd exon. Dashed lines indicate those 4 deleted nucleotides. **(B)** Chromographs illustrating the sequences of the wild-type *mapk3* sequence and the mutant allele containing a 4-nucleotide deletion. **(C)** Representative images of the heart region of the *vmhcl*$^{e13/e13}$, *mapk3*$^{e2\text{-}F1}$ and *vmhcl*$^{e13/e13}$;*mapk3*$^{e2/e2\text{-}F1}$ mutant fish and WT controls at 5 dpf. The black star indicates severe edema. The red star indicates mild edema. dpf, days post fertilization. Scale bar: 300 µm. **(D-E)** Quantification of the ventricular chamber volume and percent fractional shortening (FS) of *vmhcl*$^{e13/e13}$, *mapk3*$^{e2\text{-}F1}$ and *vmhcl*$^{e13/e13}$;*mapk3*$^{e2/e2\text{-}F1}$ mutant fish compared with the WT controls at 5 dpf. n=8, Data are presented as means ± s.d. One-way ANOVA was used for statistical analyses.

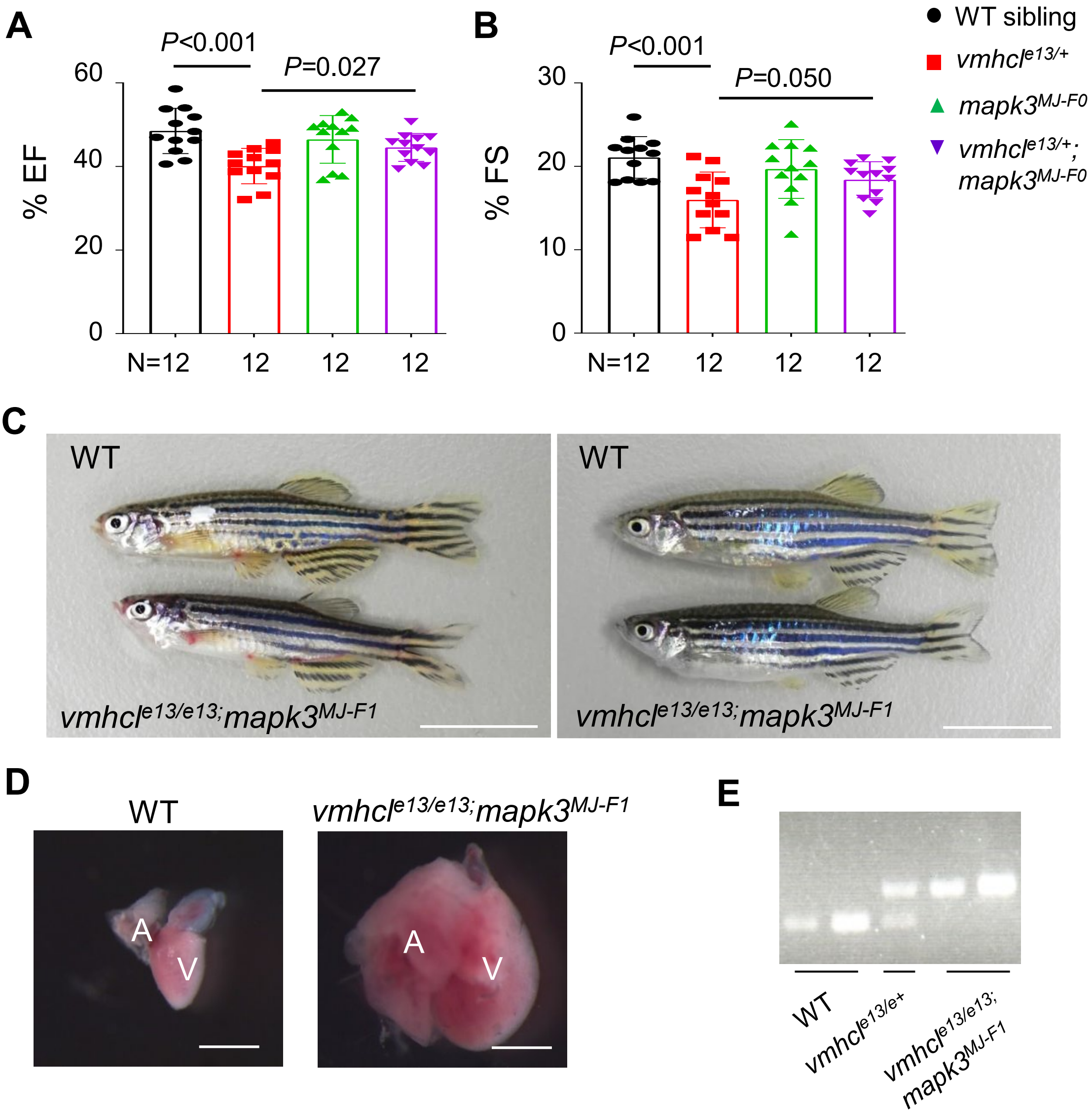

Supplemental Figure 10. Therapeutic effects of MAPK inhibition on the VAC model. (**A** and **B**): Quantification of percent ejection fraction (EF) and fraction shortening (FS) by echocardiography in fish at 9 months. n=12, Data are mean±s.d. One-way ANOVA was used for statistical analysis. **(C)** Images of the survived *vmhcl*$^{e13/e13;}$*mapk3*$^{MJ\text{-}F1}$ double mutant fish into adulthood compared with WT controls at 3 months. Scale bars, 1 cm. **(D)** Isolated hearts from one of the *vmhcl*$^{e13/e13;}$*mapk3*$^{MJ\text{-}F1}$ double mutant fish compared to WT control. Scale bar, 1 mm. V, ventricle. A, atrium. **(E)** Genotyping PCR results confirmed the homozygosity of those two survived adult *vmhcl*$^{e13/e13;}$*mapk3*$^{MJ\text{-}F1}$ double mutant fish.
